# The Six Components of Social Interactions: Actor, Partner, Relation, Activities, Context, and Evaluation

**DOI:** 10.3389/fpsyg.2021.743074

**Published:** 2022-01-10

**Authors:** Sarah Susanna Hoppler, Robin Segerer, Jana Nikitin

**Affiliations:** ^1^Department of Personality and Developmental Psychology, Faculty of Psychology, University of Basel, Basel, Switzerland; ^2^Department of Developmental and Educational Psychology, Faculty of Psychology, University of Vienna, Vienna, Austria

**Keywords:** hierarchical taxonomy, grounded theory, bottom-up approach, top-down approach, social situation, situation description, feature level

## Abstract

Social interactions are essential aspects of social relationships. Despite their centrality, there is a lack of a standardized approach to systematize social interactions. The present research developed (Study 1) and tested (Study 2) a taxonomy of social interactions. In Study 1 (5,676 descriptions of social interactions from *N* = 708 participants, age range 18–83 years), we combined a bottom-up approach based on the grounded theory with a top-down approach integrating existing empirical and theoretical literature to develop the taxonomy. The resulting taxonomy (APRACE) comprises the components Actor, Partner, Relation, Activities, Context, and Evaluation, each specified by features on three levels of abstraction. A social situation can be described by a combination of the components and their features on the respective abstraction level. Study 2 tested the APRACE using another dataset (*N* = 303, age range 18–88 years) with 1,899 descriptions of social interactions. The index scores of the six components, the frequencies of the features on the most abstract level, and their correlations were largely consistent across both studies, which supports the generalizability of the APRACE. The APRACE offers a generalizable tool for the comprehensive, parsimonious, and systematic description of social interactions and, thus, enables networked research on social interactions and application in a number of practical fields.

## Introduction

With the increase of human-machine interactions, it has become clear that the complex phenomenon of interpersonal interactions must also be made comprehensible for artificial intelligence (Devillers and Duplessis, 2017). This requires a comprehensive yet economical description of social interactions (Devillers and Duplessis, 2017). In efforts to develop such a description, the variety of social interactions between people must be considered (e.g., Krause, 1970; Moos, 1973), as must the number of different ways people perceive social interactions (e.g., King and Sorrentino, 1983; Hardin and Higgins, 1996; Gallois et al., 2005). We know surprisingly little about components that people spontaneously use and combine when they are describing their social interactions. Yet, these components might provide information about the central topics and foci of people’s social lives (Bosco et al., 2004).

**FIGURE 1:**
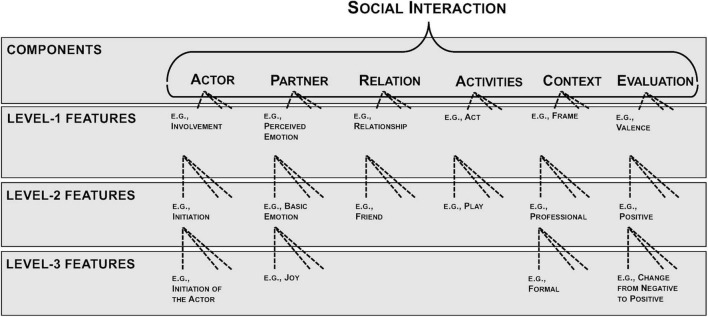
Hierarchical structure of the APRACE. The form of the display is based on Gray’s guidelines for theory mapping (Gray, 2017).

The aim of the present research is to identify subjectively meaningful components of social interactions. These interactions can be viewed as encounters between at least two people in which they attend to one another and adjust their behavior in response to one another (Reis et al., 1980). We build on the premise that people articulate relevant aspects of their lived experiences (Bosco et al., 2004), meaning that they describe parsimoniously all relevant aspects for a comprehensive understanding of an experience (Bakhtin, 1981). Consequently, descriptions of social interactions tell us what people consider meaningful as they communicate their social experiences (Grice, 1975). We use this communicative function of description to investigate which components (sets of shared characteristics) people employ to describe their social interactions. To that end, we apply a combination of a bottom-up approach according to the grounded theory (Charmaz, 2006; Heaton, 2008), and a top-down approach, integrating previous empirical and theoretical research to develop a taxonomy of social interactions (Reis, 2018). In an iterative procedure, we compare social interactions to identify conceptual content-related similarities and differences. Subsequently, we group the interaction characteristics into pre-existing or newly formulated components (see Figure 1). For example, if a person mentions the location of a social interaction, she indicates that she considers the Context component to be relevant by specifying it.

This approach is particularly expedient when developing the taxonomy of social interactions to identify their characteristics and to group them based on their level of abstraction. The resulting hierarchical structure of the taxonomy allows us to not only respect different detail levels of people’s descriptions, but also to assess how often a component appears in people’s descriptions at lower levels of abstraction. We assume that the usage frequency of a component is an indication of its relevance for a description of a social interaction. The more often a component is mentioned, the more it is in the focus of the describing person. This enables us to sum up the number of lower-level descriptions used within a component to form an index score. In this way, we obtain a weighting of the components within a description and also reveal generalizable social perception patterns across multiple data sets using correlative and typological analyses.

Given the relevance of social interactions to people’s lives (Festinger et al., 1950; Vaughan, 1986; Baumeister and Leary, 1995; Qualter et al., 2015; Tamir and Hughes, 2018), there has been some effort to describe social interactions in psychologically meaningful ways. To our knowledge, seven taxonomies of social interactions exist (Bales and Strodtbeck, 1951; Krause, 1970; Moos, 1973; Price and Blashfield, 1975; Forgas, 1976; Nascimento-Schulze, 1981; King and Sorrentino, 1983). Supplementary Table 1 gives an overview of the characteristics, procedures, number, and content of identified groups of social situations (note that we did not differentiate abstraction levels). There is a great diversity in the existing taxonomies of social interactions. Different objectives have been emphasized: Krause (1970), and King and Sorrentino (1983) have focused on specific psychological aspects, such as motivation and goal orientation; Bales (1950) and Bales and Strodtbeck (1951) on problem-solving strategies; Forgas (1976) on social episodes; Moos (1973), and Price and Blashfield (1975) on setting; and Nascimento-Schulze (1981) on face-to-face social interactions. Consequently, the identified components (originally labeled as categories; e.g., Bales and Strodtbeck, 1951) or dimensions; e.g., King and Sorrentino, 1983) of social situations vary in their conceptual meaning, such as specific activities (e.g., play, joint working; Krause, 1970), problem-solving processes (e.g., problems of orientation, problems of decision; Bales, 1950; Bales and Strodtbeck, 1951), or social environments (e.g., classrooms, family settings, psychiatric wards; Moos, 1973). The taxonomies have in common that they are not hierarchically structured, except for the taxonomy by Bales and Strodtbeck (1951) that postulates two superordinate groups of social interactions.

Along with taxonomies of social interactions, a variety of instruments are used to assess specific aspects of social interactions: *Inventory of Negative Social Interactions* (INSI; Lakey et al., 1994); *Positive and Negative Social Exchanges* (PANSE; Newsom et al., 2003, 2005); *Negative Social Interaction Scale* (NISI; Rauktis et al., 1995); *Test of Negative Social Exchange* (TENSE; Ruehlman and Karoly, 1991); and others without specific names (Schuster et al., 1990; Krause, 1995; Ingersoll-Dayton et al., 1997; Okun and Keith, 1998; Rook, 2001; Stafford et al., 2011; see Supplementary Table 2). Different from taxonomies, the aim of such instruments is less to describe social interactions comprehensively and more to assess specific characteristics of social interactions, as well as to investigate their associations with other psychological variables. Like existing taxonomies, some instruments to assess social interactions differ widely in their objectives, sample characteristics, scale creation, methods, and measured valence of a social interaction (e.g., Okun and Keith, 1998; Rook, 2001; Newsom et al., 2003, 2005) and others focus on negative social interactions only (Ruehlman and Karoly, 1991; Lakey et al., 1994; Rauktis et al., 1995).

Most of the instruments were designed for a particular purpose, rather than for broad application. Only one study has explicitly focused on the development of a general measurement instrument of social interactions (i.e., TENSE; Ruehlman and Karoly, 1991). The remaining studies pursued different goals such as investigating the relationship of social interactions with positive or negative affect, depression, or distress in social relations (e.g., Rauktis et al., 1995; Okun and Keith, 1998; Newsom et al., 2003, 2005). These different goals might account for the heterogeneity of the identified components (originally labeled as factors; e.g., Ruehlman and Karoly, 1991, or domains; e.g., Newsom et al., 2003, 2005) of social interactions. In a number of these instruments, social interactions are operationalized through the lens of social support with questions related to providing interest in well-being, favors, transportation, unwanted advice, or insensitive behavior (Schuster et al., 1990; Krause, 1995; Ingersoll-Dayton et al., 1997; Okun and Keith, 1998; Newsom et al., 2003, 2005). Only three existing questionnaires conceptualize components of social interactions more broadly (Ruehlman and Karoly, 1991; Lakey et al., 1994; Rauktis et al., 1995). Therefore, most instruments might be too specific and limited in application to capture all characteristics of a social interaction.

Most instruments are not structured in a hierarchy (e.g., Ruehlman and Karoly, 1991; Ingersoll-Dayton et al., 1997; Stafford et al., 2011). The questionnaire *TENSE* (Ruehlman and Karoly, 1991) records different kinds of negative exchanges (i.e., hostility/impatience, insensitivity, interference, and ridicule) and this focus on one aspect and neglect of others (e.g., interaction partners, motivations of interaction partners) do not allow holistic statements about concrete social situations. Those that do have a hierarchical structure differ in the number and content of their hierarchies. Krause (1995) formulates the superordinate groups negative social interactions, received support, satisfaction with support, and provided support, each divided into three to four subordinate groups. Newsom et al. (2003, 2005) postulate the superordinate groups positive social exchanges and negative social exchanges, each with four subordinate groups.

Note that besides taxonomies of social interactions, there is research on psychologically relevant characteristics of situations. This research focuses on the psychological meaning, importance and consequence of situations. Accordingly, identified dimensions of situations include psychological characteristics such as duty, intellect, adversity, demandingness, etc., (e.g., DIAMONDS, Rauthmann et al., 2014; CAPTIONS, Parrigon et al., 2017; The Situation Six, Oreg et al., 2020). In contrast, our approach is descriptive and neutral in terms of psychological meaning, enabling a broad application of the taxonomy. In addition, we develop a taxonomy of social interactions as a specific form of a situation.

To summarize, the available taxonomies and measurement tools are highly heterogeneous in terms of their concepts, hierarchization, and specificity. Due to this lack of consensus, an approach has yet to be established that describes the diversity of the social interactions as they are perceived by people (Yang et al., 2006; Saucier et al., 2007; Rauthmann et al., 2014; Meyer, 2015). To conduct research with comparable results across studies and across disciplines, however, a comprehensive but nevertheless parsimonious taxonomy of social interactions is needed (Funder, 2001; Meyer, 2015). Research on social interactions would benefit from such a generalizable and widely communicable taxonomy (Sokal, 1974; Rosch, 1978; Meyer, 2015). In the following, we aim to lay the foundation for such a taxonomy of social interactions. In order to meet the complexity of social situations ascertained in our literature research, we are targeting for a different structure than previous taxonomies. We strive to identify several specific characteristics (i.e., components) of social situations on different levels of abstraction. To capture a certain social situation, the combination of different components is necessary (e.g., Lebeuf et al., 2019). Study 1 develops the taxonomy^1^ using a combination of a bottom-up and a top-down approach. Index scores have been calculated to quantify the perceptive salience of each component in a social situation. Study 2 tests comprehensiveness and generalizability by investigating whether the quantitative properties of the model can be confirmed in a new data set of descriptions of social interactions.

## Study 1

A major concern in constructing a taxonomy of social interactions is having a high enough number of descriptions of social interactions that contain as many different social situations as possible (Pervin, 1978; Yang et al., 2006). One possible approach to generating a high number of ecologically valid descriptions of social interactions is the use of diary methods (e.g., Reis and Wheeler, 1991; Bolger et al., 2003). In their diaries, participants report what they do and feel in their daily lives on several occasions during a normal week (Csikszentmihalyi et al., 1977; Hektner et al., 2007). This method is used in Study 1 with a wide range of daily social interactions.

To develop our hierarchical taxonomy, we first apply the constant-comparative approach based on the grounded theory (Charmaz, 2006; Heaton, 2008). This bottom-up approach is based on the rationale that participants describe social interactions using their internal representations of them (Forgas, 1976; Turner, 1988); we, therefore, expect insightful information.

One shortcoming of a purely inductive bottom-up approach is that every dataset of social interactions contains only a limited sample of all possibilities. As a consequence, less frequent social interactions might not appear in the dataset (Yang et al., 2006). In addition, prevailing culture, norms, rules, and expectations influence what people experience and what they report (Forgas, 1976; Turner, 1988). A deductive top-down approach offers the opportunity to add missing information (Reis, 2018). Therefore, in a second step, we apply a top-down approach to complement characteristics identified by previous empirical and theoretical work.

To account for possibly different levels of abstraction in the descriptions, the taxonomy is structured in a hierarchy. We create this by comparing and grouping all elements of social interactions in previous research according to their content. The elements that are similar in content are the building blocks of a higher order in the hierarchy. In doing so, we aim to achieve the highest possible generalizability of the resulting taxonomy. This integrative (i.e., bottom-up and top-down) approach promises a generally accepted and broadly applicable taxonomy of social situations (Frederiksen, 1972; Meyer, 2015).

### Method

#### Sample

Study 1 uses an existing dataset with descriptions of social interactions (Nikitin and Freund, 2018). It contains records collected over the course of 1 week where participants were asked to describe their most positive and most negative social interaction of each day in an open-ended response format. The participants were recruited from all over Germany in May 2014 via a German online recruitment service with a databank of 100,000 potential respondents. The final sample comprises *N* = 744 participants between 18 and 83 years old (*M* = 49.28 years, *SD* = 16.50 years). Among them, 48.3% are female (for further sociodemographic information, see Nikitin and Freund, 2018). The study conforms to the guidelines by the local ethics committee and is considered exempt from formal ethical review.

The final data pool after the selection of valid descriptions of social interactions includes 5,676 descriptions of social interactions (60.0% positive; 40.0% negative; from *N* = 708 subjects). Approximately half of the participants are female (49.6%) and 50.4% are male. The age of the participants ranges between 18 and 83 years (*M* = 49.8 years, *SD* = 16.4 years). Concerning relationship status, 19.3% of the participants report being single, 47.1% married, 18.3% in a committed relationship, 10.3% divorced, and 5.2% widowed. More than a half of the participants (59.8%) have had children. With respect to highest level of education, 29.2% report university, 9.3% university of applied sciences, 20.2% high school, 28.8% vocational training, and 7.3% primary school (5.2% report some other education).

#### Data Collection

On seven consecutive days, participants were invited to think about the most positive and the most negative social interaction in the last 24 h and to describe them in written form as follows: “Please think briefly now of the most positive/negative social situation that you have experienced in the past 24 h. Please describe the situation in a short sentence.” Social interaction was defined as any encounter with at least one other person in which the people attend to one another and adjust their behavior in response to one another (Reis et al., 1980). To mitigate range restrictions in the selection of reported interactions due to evaluative biases, such as the negativity bias (Rozin and Royzman, 2001), subjects were explicitly asked to provide both one positive and one negative description per day. In addition to the open-format request to describe the social interactions, closed-format questions describing stable characteristics of the social interaction partners and their relationships (e.g., emotional closeness) were asked and assessed. These items are not part of the present research.

The initial dataset comprises 10,416 descriptions of social interactions (*N* = 744), with 19.4% missing descriptions. Descriptions that did not involve social interactions (e.g., “alone,” “I’m thinking,” meaningless sequences of letters) were also coded as missing (26.8%). In a small number of descriptions (0.7%), participants reported more than one social interaction within one description. These descriptions were split up accordingly. Thus, the taxonomy was developed based on 5,676 descriptions of social interactions with 3,407 positive (60%) and 2,269 negative (40%) from *N* = 708 participants with an average of *M* = 8.0 descriptions (*SD* = 4.1) per participant.

#### Statistical Data Analyses

##### Qualitative Analyses

A qualitative procedure according to the grounded theory (Charmaz, 2006; Heaton, 2008) was applied to scrutinize the taxonomy of social interactions. In a bottom-up approach, the taxonomy was designed based on a randomly drawn subsample of social interactions (N = 274 with 1,205 positive and 917 negative social interactions) to set up a first framework. In a constant comparative approach as adapted for secondary analysis of a dataset (Charmaz, 2006; Heaton, 2008), every described social interaction of the subsample was continually compared with all other described social interactions in an iterative process and similar social interactions were assigned to content-related groups of social interactions (initial and focused coding; Charmaz, 2006). Due to the large overlap of groups between the positive and negative social interactions, they were collapsed.

We then applied a top-down approach to ensure the comprehensiveness of the taxonomy and to hierarchically organize the groups of social interactions (Reis, 2018). To obtain a comprehensive taxonomy, elements of previous research were aligned with the groups of social interactions identified in the bottom-up approach. As specified in Supplementary Table 3, we detected nearly all elements from previous research in our dataset. Elements that were not detected in the dataset were added to the taxonomy.

We then organized the received groups of social interactions in a hierarchical structure. All elements of social interactions from previous empirical studies and theoretical work were compared and grouped according to their content. Elements similar in content were grouped in meaningful higher levels and finally organized into a hierarchy. This resulted in six overarching components with features on three levels representing different levels of abstraction in the hierarchical structure. Components constitute basic building blocks of social interactions and delineate broad thematic components. Features on level 1 are independent thematic areas that characterize a component. Level-2 features represent specific manifestations of level-1 features and can be further divided into features on level 3 when the content complexity is high.

Two independent raters used the resulting taxonomy to code the entire dataset. In the training phase, the raters coded 28 randomly drawn descriptions of social interactions from the dataset together. In the case of disagreement, discussions were held until there was full consensus on the coding. In the coding phase, all descriptions (including the 28 descriptions from the training phase) were randomly assigned to the two raters who independently coded 50% of the descriptions of social interactions (2,838 for each rater). To calculate interrater reliability, 550 social interactions (9.7%) were rated by both raters. Each level-1 feature was assessed as to whether it was present in the description or not. The interrater reliability of the level-1 features ranges between κ = 0.83 and κ = 0.98 (*M*_κ_ = 0.94, *SD*_κ_ = 0.03).

##### Quantitative Analyses

For social interaction descriptions with many components or level-1 features, each individual component/level-1 feature was weighted less than it was in descriptions with fewer components/level-1 features. Therefore, the quantitative analyses were based on index scores that relativize the frequencies of the mentioned components or level-1 features. For each component, the number of coded level-1 features within was divided by the number of coded level-1 features across all components. For level-1 features, the number of a coded level-1 feature was divided by the number of coded level-1 features across all components. The measurement model underlying these index scores is formative, which assumes that the lower level indicators collectively determine the meaning of the higher level construct (Avila et al., 2015).

Subsequently, we conducted quantitative statistical analyses to consider the nested data structure within participants. According to Bliese (1998), nested data should be assumed if the intraclass correlation is higher than ICC = 0.01. Due to large intraclass correlations in our data (ICC = 0.09–0.15), person effects were considered for the calculations. To account for the multilevel data structure in the frequency analysis, we counted the minimal and maximum frequencies of mentioning the corresponding component or level-1 feature within participants. Multilevel correlations were conducted with the correlation package (Makowski et al., 2020) in Rstudio (R Core Team, 2016) to control the correlations at the participant level. Multilevel correlations were calculated to discuss possible adjustments to the taxonomy, such as whether components or level-1 features should be merged or whether level-1 features should be part of another component.

### Results

#### Qualitative Analyses

##### Components

The final resulting structure of the taxonomy is shown in Figure 1^2^. On the highest level, we identified six components of social interactions that lead to the name of the taxonomy: Actor, Partner, Relation, Activities, Context, and Evaluation (APRACE). Note that the entire taxonomy is developed from the perspective of the actor, meaning that the components reflect the perception of the person describing the social interaction. Depending on the component, the description is more (e.g., Relation, Evaluation) or less (e.g., Activities, Context) subjectively influenced.

According to the common definition of social interactions (Reis et al., 1980) two parts are required for an encounter, a person and at least one other person. Therefore, the participants of a social interaction are depicted by two separate components, Actor and Partner(s). The Relation component describes the bond between the actor and the partner(s), which can affect or be affected by the perception of the social interaction (Horwitz et al., 1997; Stafford et al., 2011). During a social interaction the behaviors of the interaction partners refer to each other (Reis et al., 1980). These interdependent behaviors in the form of conversations and/or actions are represented in the component Activities. The interaction partners’ activities are embedded in a certain context (Context component). Since social interactions are constantly and automatically evaluated (Duckworth et al., 2002), the Evaluation component is an arguable part of the social interaction.

##### Features on Three Different Abstraction Levels

The six components consist of up to 10 level-1 features, which incorporate up to 18 level-2 features, which are further divided into up to 11 level-3 features. The Actor component consists of Socio-Demographic Features (such as Age and Gender) and subjective states of the actor (such as Emotion and Motivation). Parallel to the Actor component, the Partner component includes the Socio-Demographic Features and perceived states of the partner by the actor. The component Relation describes the relation between the actor and each interaction partner in terms of Relationship, Dominance, and Closeness. The interaction parties’ actions are specified in the component Activities. This component contains the Acts (such as Communication or Consumption) and Interaction Mode (such as Oral Communication, Written Communication, and Non-verbal Communication). The component Context is described by the Location, Setting, and Duration. The entire social interaction is evaluated by the actor, which is represented in the component Evaluation (such as Valence).

To describe a specific social situation with the taxonomy, features on the three different levels are combined. This integration jointly describes different kinds of social situations and yield meaning. Table 1 shows such coding examples of social interactions drawn from the sampling pool. They indicate that the level of detail differs from case to case. The more detailed the description, the more informative the coding.

**TABLE 1:** Three coding examples with the APRACE.

Description	Component	Level-1 feature	Level-2 feature	Level-3 feature
(1) “Phone call.”	Actor	Involvement	Perspective	Participant
	Partner	Dyadic or Group Contact	Dyadic Contact	
	Activities	Act	Communication	
		Interaction Mode	Oral	Media
		Physical Contact	Without	
(2) “Car driver gave me the finger.”	Actor	Involvement	Perspective	Participant
		Involvement	Initiation	Partner
	Partner	Dyadic or Group Contact	Dyadic Contact	
		Age Perceived Emotion	Adult Basic Emotion	Anger
	Relation	Relationship	Unknown Person	
	Activities	Interaction Mode Physical Contact	Non-verbal Without	Gestures
	Context	Location	Public Place/Street/Traffic	
(3) “A new girl came into my learning support group. I was able to introduce her well and although she was scared and tense in the beginning, she was very relaxed in the end, laughed and was confident.”	Actor	Involvement	Perspective	Participant
		Involvement	Initiation	Actor
	Partner	Dyadic or Group Contact	Group Contact	
		Sex	Female	
		Age	Minor	
		Perceived Motivation Perceived Emotion	Esteem Needs Basic Emotion	Joy
	Relation	Relationship	Unknown Person	
		Dominance	Subordinate	
	Activities	Act	Production	Mental
		Interaction Mode	Oral	Face-to-Face
	Context	Event	Course	
		Frame	Professional	Formal
	Evaluation	Positive	Change from Negative to Positive	

*Original descriptions of social interactions in Study 1 are coded. The assessment of whether a component is described or not is based solely on the explicit statements of the participants. Each level-1 feature has been rated on the further, lower hierarchical levels features at level 2, and features at level 3. Unmentioned level-1 features, level-2 features, and level-3 features are not listed.*

#### Quantitative Analyses

An overview of the descriptive statistics of the relative frequencies for each component and level-1 feature is shown in Table 2. The most frequently mentioned category was Context, followed by Partner, Activities, Relation, Actor, and Evaluation. The components correlated negatively or were uncorrelated, except for the positive correlation of Partner and Relation (see Table 3). The correlations among the level-1 features were between *r* = −0.28 (*p* < 0.001) and *r* = 0.51 (*p* < 0.001). With regard to the correlation strength according to Cohen (1988), the correlation between Dyadic or Group Contact and Socio-Demographic Features of the Partner component (*r* = 0.51, *p* < 0.001) and the correlation between Act and Quality of the component Activities (*r* = 0.51, *p* < 0.001) were high.

**TABLE 2:** Frequencies of the components and level-1 features mentioned in the descriptions of social interactions in Study 1 and Study 2.

Component	Level-1 feature	Relative frequency			
		Study 1 (*N* = 5,676)		Study 2 (*N* = 1,899)	
		*Md* in %	*IQR* in %	*Md* in %	*IQR* in %
Actor		<1	9.09	<1	<1
	Socio-Demographic Features	<1	<1	44.44	23.81
	Involvement	<1	<1	16.67	13.89
	Motivation	<1	<1	11.11	5.95
	Emotion	<1	<1	<1	<1
Partner		22.22	30	8.33	12.5
	Dyadic or Group Contact	11.11	14.29	<1	<1
	Socio-Demographic Features	8.33	12.5	25	25.25
	Perceived Motivation	<1	<1	<1	10
	Perceived Emotion	<1	<1	<1	<1
Relation		12.5	16.67	6.25	10
	Relationship	11.11	12.29	<1	<1
	Dominance	<1	<1	11.11	6.59
	Closeness	<1	<1	<1	<1
Activities		14.29	30	<1	<1
	Act	9.09	14.29	<1	11.11
	Interaction Mode	<1	<1	<1	<1
	Physical Contact	<1	<1	<1	<1
	Quality	<1	<1	<1	<1
	Anticipation	<1	<1	<1	<1
Context		37.5	37.5	<1	<1
	Location	<1	7.69	<1	<1
	Event	<1	<1	<1	<1
	Setting	<1	11.11	6.25	10
	Surroundings	<1	<1	<1	<1
	Frame	12.5	7.58	11.11	6.59
	Day time	<1	<1	<1	<1
	Time since last Interaction	<1	<1	<1	<1
	Duration	8.33	12.5	<1	11.11
	Time Focus	<1	<1	<1	<1
	Course	<1	<1	<1	<1
Evaluation		<1	<1	<1	<1
	Desirability	<1	<1	<1	<1
	Valence	<1	<1	<1	<1
	Fulfilment of Expectations	<1	<1	<1	<1

*Md and IQR are used to represent median and interquartile ranges of the relative frequencies of components and level-1 features within descriptions. In Study 1, the range of mentioning the corresponding component or level-1 feature within participants lies between no mention and the mention in any given description for all components and for most level-1 features (75.9%), occasionally not in the level-1 features Socio-Demographic Features (0–16.7%) and Involvement (0–30.8%) of the component Actor, Physical Contact (0–50%) of the component Activities, Day Time (66.7%), Time since last Interaction (0–50%) and Course (0–50%) of the component Context, and Fulfilment of Expectations (0–50%) of the component Evaluation. In Study 2, the range of mentioning the corresponding component or level-1 feature within participants lies between no mention and the mention in any given description for most components (83.3%) and for most level-1 features (62.1%), occasionally not in the components Activities (12.5–100%) and Evaluation (0–57.1%), in the level-1 features Socio-Demographic Features (0–16.7%) and Emotion (0–50%) of the component Actor, Perceived Emotion (0–50%) of the component Partner, Dominance (0–66.7%) and Closeness (0–28.6%) of the component Relation, Act (12.5–100%) of the component Activities, Day Time (66.7%), Time since last Interaction (0–25%) and Course (0–16.7%) of the component Context, and Desirability (0–57.1%), Valence (0–50%) and Fulfilment of Expectation (0–25%) of the component Evaluation.*

**TABLE 3:** Multilevel correlations with confidence intervals of the components in Study 1 with the relative frequencies within descriptions of components.

	1	2	3	4	5
1. Actor					
2. Partner	0.01 [−0.01, 0.04]				
3. Relation	−0.10*** [−0.13, −0.08]	0.45*** [0.43, 0.47]			
4. Activities	−0.27*** [−0.29, −0.24]	−0.19*** [−0.22, −0.17]	−0.28*** [−0.30, −0.25]		
5. Context	−0.16*** [−0.18, −0.13]	−0.40*** [−0.42, −0.38]	−0.19*** [−0.22, −0.17]	−0.36*** [−0.38, −0.33]	
6. Evaluation	−0.03* [−0.06, 0.00]	−0.13*** [−0.16, −0.11]	−0.15*** [−0.18, −0.13]	−0.09*** [−0.11, −0.06]	−0.17*** [−0.19, −0.14]

The poisson distributed variables, namely the relative frequencies of the components Actor, Partner, Relation, Activities, and Evaluation, have been transformed using an Anscombe transformation to calculate the multilevel correlations. Values in square brackets indicate the 95% confidence interval for each correlation. *p < 0.05, ***p < 0.001. The p-value of the non-significant correlation of the components Actor and Partner is p = 0.29.

### Discussion

In Study 1, we combined a bottom-up with a top-down approach and developed a comprehensive taxonomy of social interactions (Reis, 2018). In their descriptions of social interactions, participants used the six components Actor, Partner, Activities, Relation, Context, and Evaluation. The results of the component frequency order can be interpreted as the way context information is required to understand and reconstruct a majority of social interactions. This interpretation is based on the above outlined assumption that all speech has a communicative function (Bakhtin, 1981; Bosco et al., 2004). The most important aspects in describing social interactions, based on level-1 features, appear to be the frame of the social interaction, what has been done in the situation, and number of people involved.

Whether a social interaction was perceived as positive or negative (Evaluation: <1%) was least mentioned. The most obvious explanation is that the participants were explicitly requested in the diary to describe their most positive and most negative social interaction and, thus, did not consider it necessary to mention the valence of the interaction in the description. Another explanation is that when people describe a social interaction, they implicitly communicate the valence of the interaction in the description of the action (e.g., when they report that they had an argument, they might assume that it indicates negative valence of the interaction).

Within the correlation matrices of the components, the highest correlation was found between Partner and Relation. This could be partly due to a linguistic artifact. If, for example, the level-1 feature Relationship of the component Relation is mentioned, in some cases the gender of the interaction partner, which belongs to the level-1 feature Socio-Demographic Features of the component Partner, is syntactically marked via gender morphemes. Thus, based on one German word like “Freundin,” two substantially different features are captured. Since gender morphemes are less prevalent in other languages, like English (e.g., friend, colleague, roommate, neighbor, boss), one might assume that the positive correlation between the components Partner and Relation might not be a universal phenomenon and the two components should not be merged.

The correlation analyses of level-1 features showed that most features should be treated separately. Those features that were highly correlated (according to Cohen, 1988), differed either in terms of content (e.g., Act and Quality of Activities) or were based at least partially on a linguistic artifact (e.g., by naming the interaction partner in singular, such as “eating with my wife,” the participant provide information both on the feature Gender and Dyadic Contact of the component Partner), and should thus remain independent. The level-1 features that were similarly correlated with two components are better described as part of the presumed component (e.g., Body Contact was correlated similarly with the components Activities and Partner but it better describes what is being done than the characteristic of the partner).

One limitation of Study 1 is that the participants were requested to describe their most positive and most negative social interaction of the day and, thus, possibly did not consider it necessary to mention the valence of the interaction in their descriptions. In addition, describing the most positive and the most negative interaction of the day might lead to a selection of particularly salient interactions and underrepresentation of interactions that are more mundane. To address these possible shortcomings and test the developed taxonomy on another data set, we conducted a second study that used a different sampling criterion for the descriptions of social interactions wherein participants were asked to describe the last social interaction they had experienced.

## Study 2

In Study 2 we implemented the APRACE on another dataset to test both its generalizability and its comprehensiveness. From two independent raters, the final formulated components of the taxonomy of social interactions were applied to a subsample of a diary study (Nikitin and Freund, 2021). Different from Study 1, participants were requested to describe their last social interaction, not the most positive and most negative social interactions of the day. Thus, Study 2 enables us to test the prevalence of Study 1 components within unguided descriptions of social interactions. In general, we use the dataset from Study 2 to explore whether the distribution patterns and correlations found in Study 1 could be replicated in another dataset.

### Method

#### Sample

The study was conducted with the approval of the local ethics committee for psychological and related research. A total of *N* = 329 participants (*M* = 46.77 years, *SD* = 20.27 years, range 18--88 years; 57.4% women) took part in the paper-pencil diary study. They were recruited between 2017 and 2019 via the university’s participant tools, lectures for students and seniors, and social networks of the students. Due to drop out during the study (*n* = 16), the number of participants was reduced to *N* = 313. Since the study followed an experimental design^3^ that might have altered social interactions or their descriptions, we used only 2,028 descriptions of social interactions from the baseline measurement with no experimental manipulation (from *N* = 308 participants). Due to missing or illegible descriptions or descriptions that were not valid for a social interaction (e.g., “working alone”), 129 descriptions from 69 participants were classified as missing. The final data set comprises 1,899 diary entries with descriptions of social interactions from *N* = 303 participants, with an average of 7.7 diary entries per participant (*SD* = 0.7 diary entries, range 4–9 diary entries). In this remaining sample, participants were 18 to 88 years old (*M* = 46.6 years, *SD* = 20.3 years). Of the participants, 57.4% were female. Regarding relationship status, 25.6% of the participants were single, 31.6% were married, 26.6% were in a stable relationship, 2.7% were in an open relationship, 9.3% were divorced or separated, and 3.3% were widowed. Less than half of the participants (40.5%) had children. With respect to highest level of education, 26.2% reported university, 29.6% high school, 40.6% vocational training, 2.7% primary school, and 1% reported some other education.

#### Study Procedure

Participants received instructions for the diary study in an individual laboratory session. Subsequently, participants received eight signals of an alarm watch during the day (every 90 min starting individually 1.5 h after waking). The signals remined the participants to describe their last social interaction (“Please describe your last social interaction in a short sentence.”). After each description, participants were asked to describe the interaction partner, their motivation, and their emotions during the interaction in a closed response format. These items are not part of the present research.

#### Implementation of the Six-Categorical Taxonomy of Social Interactions

The APRACE was used by two research assistants independently to code to all 1,899 descriptions of social interactions. The procedure consisted of the following two phases:

##### Training Phase

The first author introduced two research assistants to the definitions of the six components^4^. After the familiarization, the two raters independently coded a total of 72 randomly drawn descriptions of social interactions (3.8% of the total number). In subsequent meetings between the two raters and the first author, the coding was compared and – in the case of diverging codes – discussed until a final agreement was achieved.

##### Coding Phase

The two raters then separately coded 50% of the descriptions of social interactions (949 and 950 randomly assigned descriptions to the raters). To examine interrater reliability, 198 descriptions of social interactions (10.4% of the total number) were coded by both raters. Comparing the level-1 features, the interrater reliability ranges between κ = 0.32 and κ = 1 (*M*_κ_ = 0.8, *SD*_κ_ = 0.16).

### Results

Due to large intraclass correlations (ICC = 0.07–0.18), person effects were considered for the calculations (Bliese, 1998).

#### Qualitative Analyses

Every description of a social interaction could be assigned to at least one of the 29 level-1 features. No description required a further component or a further feature at any of the levels.

#### Quantitative Analyses

Table 2 gives an overview of the components‘ relative frequencies. The median scores of the six components showed nearly the same rank order of the relative frequencies (*rho* = 0.81, *p* = 0.05) as in Study 1. Only the component Activities ranked differently. In Study 1, Activities followed the Partner component, whereas in Study 2, the component Activities took the first position. The rankings of the median scores of the 29 level-1 features were very similar in Study 1 and Study 2 (*rho* = 0.69, *p* < 0.001). The most distinct ranks resulted for the level-1 feature Interaction Mode of the Activities component, and for Act of the Activities component, which ranked highest in Study 2. Table 4 shows that the components correlated negatively or weakly positively with each other, except for the strong positive correlation between the components Partner and Relation. Study 1 and Study 2 showed very similar ranks of these correlations at the level of the components (*rho* = 0.83, *p* < 0.01) and of the level-1 features (*rho* = 0.60, *p* < 0.001).

**TABLE 4:** Multilevel correlations with confidence intervals of the components in Study 2 with the relative frequencies within descriptions of components.

	1	2	3	4	5
1. Actor					
2. Partner	0.06* [0.01, 0.10]				
3. Relation	−0.10*** [−0.15, −0.06]	0.55*** [0.51, 0.58]			
4. Activities	−0.26*** [−0.30, −0.22]	−0.40*** [−0.44, −0.36]	−0.37*** [−0.41, −0.33]		
5. Context	0.005 [−0.04, 0.05]	−0.31*** [−0.35, −0.27]	−0.21*** [−0.25, −0.17]	−0.58*** [−0.61, −0.55]	
6. Evaluation	0.06** [0.02, 0.011]	−0.06** [−0.11, −0.02]	−0.06** [−0.11, −0.02]	−0.13*** [−0.17, −0.08]	0.06** [0.02, 011]

The poisson distributed variables, namely the relative frequencies of the components Actor, Partner, Relation, Context, and Evaluation, have been transformed using an Anscombe transformation to calculate the multilevel correlations. Values in square brackets indicate the 95% confidence interval for each correlation. *p < 0.05, **p < 0.01, ***p < 0.001. The p-value of the non-significant correlation of the components Actor and Context is p = 0.85.

### Discussion

In Study 2, we implemented the newly developed taxonomy with another dataset in order to further test its applicability and to replicate the quantitative findings from Study 1. Comparing the descriptive scores and correlations, Study 2 supported the results of Study 1. Although the frequencies, the distribution of the index scores, and the correlations differed in numbers between Study 1 and 2, the ranking patterns were very similar in each of these parameters. Only the component Activities ranked differently. In Study 1, the Activities component was the third most frequent – following the components Context and Partner – whereas in Study 2 it was the most frequent component – followed by the components Context and Partner. The inspection of the level-1 features indicates that this is due in particular to the features Act and Interaction Mode of the component Activities. The reason for the different rankings might be the different time windows in which the interactions were assessed in Study 2 (hours) compared to Study 1 (days). Activities might change more quickly than interaction partners within hours, which might result in higher heterogeneity of the Activity component in Study 2. In contrast, reflecting on all social interactions at the end of the day, as in Study 1, might bring the different interaction partners more into focus, and here the component Partner ranks second. In both studies, some features of the social interactions were assessed in addition to the open-format descriptions (i.e., socio-demographic features of the actor, closeness to the interaction partner, and valence in Study 1; socio-demographics of the actor and partner, relationship and closeness to the interaction partner, initiation, motivation, frame, and valence in Study 2). The questions about the frame relating to private or professional interactions as context features were asked in Study 2, but not in Study 1. Since the participants might have noticed that context features were queried elsewhere, they may not have repeated this information in their descriptions. This might explain why the Context component was ranked second in Study 2 and first in Study 1. Despite this one exception, very similar patterns emerged with respect to the frequencies of the level-1 features, the distributions of index scores, and the correlations among index scores in Study 1 and Study 2. This is remarkable given the different study designs. It can be concluded that descriptions of social situations are relatively similar in different samples, different temporal settings, and with different instructions.

## General Discussion

In the present work, we have developed an approach to describe social interactions. The APRACE consists of the six components Actor, Partner, Relation, Activities, Context, and Evaluation. They each incorporate features on different levels of abstraction, which in their combination jointly yield meaning. Across two datasets, the frequencies of the level-1 features and the distributions and correlations of the index scores of the individual components have shown satisfactory consistency. Thus, the APRACE, despite its relative parsimony with the possibility to describe a social interaction based on one or just a few features, can depict all conceivable sorts of situations of social interactions, irrespective of whether they are described abstractly or in great detail. Below, we discuss the taxonomy in comparison to existing tools, identify its strengths and limitations, and outline its possible applications as well as directions for future research.

The strength of the present approach compared to existing taxonomies and measurement tools is a combination bottom-up and top-down approach that enables us to include different sources of information on social interactions (Reis, 2018). In contrast to previous taxonomies and measurement tools, the APRACE was developed based on people’s daily lives with no theoretical restrictions. In addition, the relatively gender-balanced samples with participants of a wide age range that were used in the present research are unique. This might explain why our taxonomy does not replicate any of the existing classifications of social interactions. However, the elements of existing taxonomies and instruments are included in our taxonomy, supporting its comprehensiveness. Further, many of the existing taxonomies and measurement tools had been developed decades ago. Indeed, the most comparable study to our approach was conducted almost 50 years ago (Forgas, 1976).

We developed the APRACE using a combination of a data-based bottom-up and a top-down approach based on social psychological research or from related social sciences. If we compare the components of the APRACE with research results from other disciplines, we see parallels to work from human brain research. In the Constraint Satisfaction Model (Miller et al., 2019), the scenario level with the slots “Who,” “Does what,” “To whom,” “Where/When,” “With what effect,” and “Why” is important to construct social meaning. These slots correspond to the components of the APRACE. Thus, through this link to neurocognitive findings, the APRACE finds interdisciplinary support.

As adult social development takes place in a specific sociohistorical context (Bühler and Nikitin, 2020), cohort effects might have an impact on the descriptions. The APRACE is a product of a particular time and place. Although we invested the greatest possible effort to develop a comprehensive taxonomy, researchers in other cultural or historical contexts might identify further or different features of social interactions. Thus, the APRACE is open for comments and additions by other researchers (i.e., expert validation; Lincoln and Guba, 1985; Flick, 2019). For example, it might be necessary to supplement the specifiers at lower hierarchical levels. Nevertheless, the present taxonomy of social interactions is carefully balanced in terms of detail and parsimony. Even without specific adaptations, it provides a useful framework to document and quantify social interactions.

Despite the comprehensiveness of the APRACE, the question remains how representative are the descriptions of social interactions with which it was developed and tested. Obviously, when participants are asked to describe their most positive and negative social interaction of the day (as in Study 1) or the last social interaction within 1.5 h (as in Study 2), many social interactions – particularly when they are relatively rare or subtle – might remain undetected. Apart from the fact that any taxonomy of social interactions is likely to remain incomplete, the present research indicates that the developed components and features of the taxonomy can be identified across at least two different samples with similar frequencies and correlation patterns. These results are in support of the APRACE.

Our coding was relatively conservative, meaning we coded only unambiguous information and avoided any subjective interpretation (Saucier et al., 2007). The strength of this procedure is its high objectivity, which is reflected in the robust interrater reliability of the coding. The limitation of this procedure is that meaningful information might be missed. This is particularly the case when the level of detail and concreteness in the descriptions is low. Encouraging participants to describe their social interactions in more detail can counteract this possible loss of information in the coding.

Some features of the social interactions were assessed using closed-format questions, in addition to the open-format descriptions, in both studies (e.g., closeness to the interaction partner, valence of the interaction). Since the participants knew that this information would be assessed elsewhere, they might not have reiterated it in their descriptions of the social interactions. This might be a reason why the component Evaluation was the least frequently used component in both studies (as it was assessed in additional, closed-format questions). A study design assessing only descriptions of social interactions without additional information may result in a different frequency order of the six components. Nevertheless, even features that were assessed in addition to the descriptions of social interactions were also detected in the descriptions. This double referencing indicates the communicative importance of these features (Bakhtin, 1981).

As a next step, social situations might be examined in more depth. For example, patterns of frequent combinations of different features might be explored. By involving additional variables (e.g., age, gender, well-being in the interaction), the social meaning of such conceptually different patterns might be clarified. The APRACE further allows to investigate important questions about the role of social context for people’s lives. Many effects of social context factors on human development over the lifespan are not yet fully understood. Despite its long theoretical tradition (e.g., Bronfenbrenner et al., 1981; Bronfenbrenner, 1994), knowledge about which social context factors and which characteristics of social interactions shape development is still relatively scarce. For example, more research is needed on aspects of social interactions that fulfill age-specific functions (e.g., Havighurst, 1963; Carstensen et al., 1999), fit specific personality traits (Wrzus et al., 2016), are related to psychological meaning (e.g., Rauthmann et al., 2014; Parrigon et al., 2017; Oreg et al., 2020), or are associated with mental disorders (Borsboom, 2017). Currently – in the face of the COVID-19 pandemic – the question arises as to how situations of social interactions change under the behavioral rules of social distancing (De Quervain et al., 2020). Also, a variety of practical implications are possible. The APRACE can be a starting point in programming care and serving robots to act appropriately in interactions with humans (Devillers and Duplessis, 2017). Organizational psychology might apply the APRACE to find a person’s fit to specific professional contexts (Wrzus et al., 2016). In psychotherapy, the APRACE might complement traditional behavioral analysis.

In conclusion, the APRACE is a comprehensive and parsimonious description instrument of social interactions that provides a common language for the depiction of social interactions. It is flexible and universal and can be applied to different areas in research and practice.

## Data Availability Statement

The original contributions presented in the study are included in the article/Supplementary Material, further inquiries can be directed to the corresponding author.

## Ethics Statement

The studies involving human participants were reviewed and approved by the Local Ethics Committee for Psychological and Related Research of the University of Zurich. The patients/participants provided their written informed consent to participate in this study.

## Author Contributions

SH contributed to the data collection, the development of the taxonomy of social interactions, the data analysis, and interpretation of the data and wrote the first draft of the manuscript. RS critically revised the manuscript and contributed with substantial statistical content. JN contributed to the design of the studies, the data collection and analysis, and interpretation of the data and critically revised the manuscript with substantial contributions of intellectual content. All authors contributed to the article and approved the submitted version.

## Conflict of Interest

The authors declare that the research was conducted in the absence of any commercial or financial relationships that could be construed as a potential conflict of interest.

## Publisher’s Note

All claims expressed in this article are solely those of the authors and do not necessarily represent those of their affiliated organizations, or those of the publisher, the editors and the reviewers. Any product that may be evaluated in this article, or claim that may be made by its manufacturer, is not guaranteed or endorsed by the publisher.

## Footnotes

1 We do not use the term “taxonomy” in a biological sense with the aim of individual categorizations in a classification system, but as a description system similar to Thornton and Tamir (2021), as it is common in psychological research.

2 Definitions and a detailed list of all components and the features on the three different abstraction levels as well as the literature that was the basis for the top-down approach are reported in the Supplementary Material (see Supplementary Table 4).

3 As an experimental manipulation, participants were instructed to focus on others or on themselves during social interaction.

4 These definitions are the same as for the development of the APRACE. All definitions are provided in the Supplementary Material (see Supplementary Table 4).
