# Supplementary material for: The Six Components of Social Interactions: Actor, Partner, Relation, Activities, Context, and Evaluation
Source: Front Psychol. 2022 Jan 10;12:743074. doi: 10.3389/fpsyg.2021.743074 (PMC8784599; doi:10.3389/fpsyg.2021.743074)
Supplement: Supplementary file 1 [file Data_Sheet_1.PDF]

Supplementary Material

Table S1

Existing Taxonomies of Social Interactions

Authors	*Study Objectives*	*Participants*	*Sampling Pool Generation*	*Procedure and Analysis*	*N of Identified Groups of Social Interactions*	*Content of Identified Groups of Social Interactions*
Bales (1950); Bales & Strodtbeck (1951)	Phases in group problem solving in work context	–	–	–	6	Task area: problems of orientation, evaluation, and control<br>Socio-emotional area: problems of decision, tension-management, and integration
Krause (1970)	Motivation in social situations	–	Traditional classes of social situations in every culture	Theoretical work	7	play, joint working, trading, fighting, sponsored teaching, serving, self-disclosure
Moos (1973)	Social institutions	–	Theoretical work on six dimensions of social environments	Similarity	10	psychiatric wards, community-oriented psychiatric treatment programs, correctional institutions, military basic training, university student residences, classrooms, social, task-oriented and therapeutic groups, work milieus, families
Price & Blashfield (1975)	Behavior settings	Unspecified, a small midwestern town taken from Barker (1968/9)	Salient characteristics of behavior settings taken from Barker (1968/9): authority system, action patterns, member, performer, target, number of individuals, duration, frequency	Situational features; principal component analysis; cluster analysis	8	Local business setting; religious vs. government settings; youth performance settings; elementary school settings; high school settings; adult settings; women`s organizational settings; men`s organizational settings
Forgas (1976)	Social episodes in two different milieus	Students and housewives (20-30 years)	Self-reported social episodes	Similarity judgment; situational features; multidimensional scaling; hierarchical cluster analysis	Students: 3<br>Housewives: 2	Involvement, pleasantness, know how to behave<br>Perceived intimacy of situations, subjective self-confidence over situations
Nascimento-Schulze (1981)	Face-to-face social interactions	Students	Researcher and student suggested face-to-face social interactions	Similarity judgment; situational features; principal component factor analysis	2	Constraints; involvement

Authors	*Study Objectives*	*Participants*	*Sampling Pool Generation*	*Procedure and Analysis*	*N of Identified Groups of Social Interactions*	*Content of Identified Groups of Social Interactions*
King & Sorrentino (1983)	Goal-oriented psychological dimensions	Students	Item writers formulated goal-oriented international situations	Similarity judgment; situational features; multidimensional scaling	7	Pleasant –unpleasant; accidentally caused/involved – intentionally caused/uninvolved; physically oriented – socially oriented; sensitive – insensitive; nonintimate – intimate; long – short duration; work- oriented – relaxation-oriented

Note. Taxonomies are listed chronologically. Groups of social interactions refer to both components and features and can be assigned to different levels of the taxonomy hierarchy.

Table S2

Existing Measurement Tools of Social Interactions

Authors	*Scope of Application*	*Study Objectives*	*Participants*	*Item Generation*	*Valence of Items*	*N of Items*	*Item Examples*	*Procedure and Statistical Analysis*	*Content of Identified Groups of Social Interactions*
Schuster et al. (1990)	Specific	Social interactions and depressed mood	Adults	Self-developed	Positive	2	How much your spouse understands the way you feel about things	Pre-defined groups of social interactions	Spouse supportive, relative supportive, friend supportive
					Negative	3	How often you have an unpleasant disagreement with your spouse		Spouse negative, relative negative, friend negative
Ruehlman & Karoly (1991)	Broad: Questionnaire, *Test of Negative Social Exchange (TENSE)*	Development and validation of the questionnaire *TENSE*	Study 1: Students Study 2: Students	Literature based items	Negative	18	Yelled at me, was inconsiderate, prevented me from working on my goals	Study 1: Factor analysis Study 2: Confirmatory factor analysis	Hostility /impatience, insensitivity, interference, ridicule
Lakey et al. (1994)	Broad: Questionnaire, *Inventory of Negative Social Interactions (INSI)*	Negative social interactions, social support, cognition, and psychological distress	Students	Self-developed, modeled after supportive transactions items of *ISSB* (Barrera et al., 1981)	Negative	40	Criticized you, asked you to do something unreasonable	Pre-defined groups of social interactions	Negative interactions
Krause (1995)	Specific	Negative interactions and satisfaction with social support	Noninstitutionalized and retired older adults	Positive items based on Krause & Markides (1990)	Positive	9	Others provide transportation, others showed interest and concern in your well-being, satisfaction with support	Factor analysis	Received support: informational support, tangible help, emotional support; Satisfaction with support: informational support, tangible help, emotional support; Support provided: informational support, tangible help, emotional support
				Self-developed negative items	Negative	4	Others make too many demands, others are pry into affairs	Pre-defined groups of social interactions	Negative interactions: demands, critical, pry, take advantage
Rauktis et al. (1995)	Specific: Questionnaire,	Negative social interactions,	Young, middle-aged, and older	Self-developed, based on a 5-	Negative	16	How often do friends or family criticize	Factor analysis	Negative social interaction

Authors	*Scope of Application*	*Study Objectives*	*Participants*	*Item Generation*	*Valence of Items*	*N of Items*	*Item Examples*	*Procedure and Statistical Analysis*	*Content of Identified Groups of Social Interactions*
	Negative Social Interaction Scale (NISI)	distress, and depression	caregiving adults	item scale by Schulz et al. (1992), guided by Rook & Pietromonaco (1987)			how you handle things with (patient)?		
Ingersoll-Dayton et al. (1997)	Specific	Social exchanges and positive and negative affect	Middle-aged and older adults	Self-developed	Positive	6	Reassurance, confiding, receiving care when ill	Pre-defined groups of social interactions	Positive exchanges
					Negative	3	How many got on their nerves, how many did not understand them		Negative exchanges
Okun & Keith (1998)	Specific	Social exchanges and depressive symptoms	Younger and older adults	Self-developed	Positive	2	Makes them feel Loved and cared for, is willing to listen when they need to talk about their worries or problems	Pre-defined groups of social interactions	Positive social exchanges
					Negative	2	Makes too many demands, is critical or them or what they do		Negative social exchanges
Rook (2001)	Specific	Social exchanges and emotional health	Older adults	Self-developed	Positive	14	Had been visited by a friend, borrowed something from someone	Pre-defined groups of social interactions	Positive daily social exchanges
					Negative	6	Behaved in an unkind manner toward them, hurt their feelings		Negative daily social exchanges
Newsom et al. (2003, 2005)	Broad: Questionnaire, *Positive and Negative Social Exchanges (PANSE)*	2003: Social exchanges and positive and negative affect<br>2005: Social exchanges and psychological health	2003: Older adults<br>2005: noninstitu-tionalized and cognitively healthy older adults	Self-developed	Positive	12	Make useful suggestions, do favors and other things for you	Focus groups, card-sorting tasks, confirmatory factor analysis	Positive domain: informational support, instrumental support, emotional support, companionship
					Negative	12	Leave you out of activities you would have enjoyed, fail to spend enough time		Negative domain: unwanted advice or intrusion, failure to provide help, unsympathetic or insensitive behavior, rejection or neglect

Authors	*Scope of Application*	*Study Objectives*	*Participants*	*Item Generation*	*Valence of Items*	*N of Items*	*Item Examples*	*Procedure and Statistical Analysis*	*Content of Identified Groups of Social Interactions*
							with you		
Stafford et al. (2011)	Specific	Social exchanges and depression	Middle-aged and older adults	Self-developed	Positive	3	Empathy, dependability, confiding	Pre-defined groups of social interactions	Positive exchanges
					Negative	3	Criticism, being let down, annoyance		Negative exchanges

Note. Measurement tools are listed chronologically. The *Inventory of Socially Supportive Behaviors* (*ISSB*; Barrera et al., 1981) was developed based on literature reviews, empirical research, and discussion articles. These groups of social interactions correspond to components and features and can be assigned to different levels of the model hierarchy. Scope of application can be broad vs. specific for a given study.

Table S3

Elements of Social Interactions in Existing Models of Social Situations, Existing Measurement Tools, Theories, Situational Classifications, Research Studies, and Lexicographic Databases

Authors	Setup	Elements of Social Interactions	Elements of Social Interactions Used for Hierarchization of our Taxonomy and its Integration	
Lewin (1936)	Theory	Person, situation	Person	Component Actor
Deci & Ryan (1985)	Theory	Competence, autonomy, relatedness	Competence, autonomy	Level-1 feature Motivation in components Actor and Partner
			Relatedness	Level-1 feature Relationship of component Relation
Turner (1988); Goffman (1974)	Theory	Sociocultural frames: institutional, organizational, Interpersonal; demographic frames: number of persons, density of persons, migration of persons; personal frames: friendship, biographic, intimate, physical frames: use of props, use of stages, use of ecology	Sociocultural frames	Level-3 feature Form in level-2 feature Private and Professional in level-1 feature Frame in component Context
			Demographic frames	Level-1 feature Dyadic or Group Contact in component Partner
			Personal frames	Component Relation
Ortony & Turner (1990); Zinck & Newen (2008)	Theory	Joy, fear, anger, sadness	None	None
Carstensen (1992); Carstensen et al. (1999)	Theory	Emotion regulation, acquisition of knowledge	None	None
Zinck & Newen (2008)	Theory	Self-referential emotions, shame, guilt, jealousy, envy, pride, love, contempt, humiliation, embarassment, hubris	Self-referential emotions	Level-2 feature of Non-basic Emotions in level-1 feature Emotion in components Actor and Partner
Fredrickson (2004)	Theory	Emotion, motivation	Emotion	Level-1 feature Emotion in components Actor and Partner
			Motivation	Level-1 feature Motivation in components Actor and Partner
Lodi-Smith & Roberts (2007); Roberts & Wood (2006); Roberts et al. (2005)	Theory	Family, work, formal religious institutions, volunteer work, partner, parents, grandparents, children, siblings, siblings` children	Family, work	Level-1 feature Frame in component Context
Magnusson (1971)	Classification of situations	Positive, negative, passive, social, active	Positive and negative	Component Evaluation
Van Heck (1984, 1989)	Classification of situations	Interpersonal conflict, joint working, intimacy, recreation, traveling, rituals, sport, excesses, serving, trading	None	None
Eckes (1995)	Classification of situations	Nonintimate, emotionally uninvolved, informal, relaxed, social, familiar social, frightening, emotionally involving, competitive		
Saucier et al. (2007)	Classification of	Sleep-related contexts, conditions of dress,	Physical locales	Level-1 feature Location in component Context

Authors	Setup	Elements of Social Interactions	Elements of Social Interactions Used for Hierarchization of our Taxonomy and its Integration	
	situations	condition of finance, physical locales, affective/emotive states, relational contexts, activities, conditions of time-pressure, goal-realization states, states of identity-realization, motivational (goal-seeking) states, states of expressing another trait, transaction-dependent contexts, cognitive states, conditions of crisis and danger	Affective/emotive states<br>Goal-realization states, motivational (goal-seeking) states<br>Activities	Level-1 feature Emotion in components Actor and Partner<br>Level-1 feature Motivation in components Actor and Partner<br>Component Activities
Sherman et al. (2012, 2013)	Classification of situations	Social situations, school work in class with others, school work at home, school work alone, recreating, getting ready for something, work, unpleasant situations	Unpleasant situations	Level-1 feature Valence in component Evaluation
Rauthmann et al. (2014)	Classification of situations	Duty, intellect, adversity, mating, positivity, negativity, deception, sociality, person aspects, social roles, states (affect, motivation), interactions, family, mate/spouse, friends, cohabitants, colleagues, objects, events, activities, sports/training, exam, preparing food, eating, drinking, communicating, computer/online, videogames, reading, working/studying, shopping, grooming, waiting, sleep, music/dance, telephone, at home, in kitchen, in bathroom, in bed, at university, in bar/café, restaurant	Positivity, negativity<br>Adversity<br>Person aspects<br>States (affect, motivation)<br>Communication<br>Interaction partner<br>Events<br>Activities<br>Places, settings	Level-1 feature Valence in component Evaluation<br>Component Evaluation<br>Component Actor<br>Level-1 feature Emotion and Motivation in components Actor and Partner<br>Level-1 feature Interaction Mode in component Activities<br>Level-1 feature Relationship in component Relation<br>Level-1 feature Event in component Context<br>Level-1 feature Act in component Activities<br>Level-1 feature Location in the component Context
Parrigon et al. (2016)	Classification of situations	Complexity, adversity, positive valence, typicality, importance, humor, negative valence	Adversity<br>Positive and negative valence	Component Evaluation<br>Level-1 feature Valence in component Evaluation
Oreg et al. (2020)	Classification of situations	Negativity, positivity, familiarity, demandingness, oddness, straightforwardness	Negativity, positivity	Level-1 feature Valence in component Evaluation
Bales (1950); Bales & Strodtbeck (1951)	Classification of social situations	Task area, problems of orientation, evaluation, control, socio-emotional area, problems of decision, tension-management, and integration	Socio-emotional area	Level-1 feature Emotion and Motivation in components Actor and Partner
Krause (1970)	Classification of social situations	Play, joint working, trading, fighting, sponsored teaching, serving, self-disclosure	None	
Moos (1973)	Classification of social situations	Classrooms, social and task-oriented groups, work milieus, families, psychiatric wards, community-oriented psychiatric treatment programs, correctional institutions, military basic training, university student residences, therapeutic groups	Work milieus<br>Families	Level-1 feature Location in component Context<br>Level-1 feature Relationship in component Relation
Price & Blashfield (1975)	Classification of social situations	Local business setting, religious and government settings, youth performance settings, elementary school settings, high school settings, adult	None	

**Authors**	**Setup**	**Elements of Social Interactions**	**Elements of Social Interactions Used for Hierarchization of our Taxonomy and its Integration**	
		settings, women`s organizational settings, men`s organizational settings		
Forgas (1976)	Classification of social situations	Involvement, pleasantness, know how to behave, perceived intimacy of situations, subjective self-confidence over situations	Pleasantness	Component Evaluation
Nascimento-Schulze (1981)	Classification of social situations	Constraints; involvement		
King & Sorrentino (1983)	Classification of social situations	Pleasant – unpleasant; accidentally caused/involved – intentionally caused/uninvolved; physically oriented – socially oriented; sensitive – insensitive; nonintimate – intimate; long – short duration; work- oriented – relaxation-oriented	Pleasant – unpleasant	Component Evaluation
Murray (1943); Wyatt (1947)	Measurement tool (questionnaire)	Level of interpretation, other figures, personal relationships, outcome, story-description, stimulus perception, deviation from typical responses, deviation from self, time trend, tone of story, quality of telling, focal figure, strivings, avoidances, presses, theme	Other figures<br>Outcome	Component Partner<br>Component Evaluation
Schuster et al. (1990)	Measurement tool	Spouse supportive, relative supportive, friend Supportive, spouse negative, relative negative, friend negative	Motivation<br>Negative interactions	Level-1 feature Motivation in component Partner<br>Component Evaluation
Reis & Wheeler (1991)	Measurement tool (questionnaire)	Sex of the interaction partner, dyadic or group contact, time, duration, emotion, valence, initiation, quality engagement, closeness in the situation, format, content, focus of conversation	Emotion<br>Valence	Level-1 feature Emotion in components Actor and Partner<br>Level-1 feature Valence in component Evaluation
Ruehlman & Karoly (1991)	Measurement tool (questionnaire)	Hostility/impatience, insensitivity, interference, ridicule	None	None
Lakey et al. (1994)	Measurement tool (questionnaire)	Negative interactions	Negative interactions	Component Evaluation
Krause (1995)	Measurement tool	Received support, informational support, tangible help, emotional support, satisfaction with support, negative interactions, demands, critical, pry, take advantage	Negative interactions	Component Evaluation
Rauktis et al. (1995)	Measurement tool (questionnaire)	Negative social interaction	Negative social interaction	Component Evaluation
Ingersoll-Dayton et al. (1997)	Measurement tool	Positive exchanges, negative exchanges	Positive exchanges, negative exchanges	Component Evaluation
Okun & Keith (1998)	Measurement tool	Positive social exchanges, negative social exchanges	Positive social exchanges, negative social exchanges	Component Evaluation
Rook (2001)	Measurement tool	Positive daily social exchanges, negative daily social exchanges	Positive daily social exchanges, negative daily social exchanges	Component Evaluation

Authors	Setup	Elements of Social Interactions	Elements of Social Interactions Used for Hierarchization of our Taxonomy and its Integration	
Newsom et al. (2003, 2005)	Measurement tool (questionnaire)	Positive components, informational support, instrumental support, emotional support, companionship, negative components, unwanted advice or intrusion, failure to provide help, unsympathetic or insensitive behavior, rejection or neglect	Positive components, negative components	Component Evaluation
Stafford et al. (2011)	Measurement tool	Positive exchanges, negative exchanges	Positive exchanges, negative exchanges	Component Evaluation
Horwitz et al. (1997), Stafford et al. (2011)	Study	Interaction partner, relation(ship)	Interaction partner relation(ship)	Level-1 feature Relationship in component Relation
Duckworth et al. (2002)	Study	Valence, process valence, outcome valence	Process valence	Level-1 feature Valence in component Evaluation
			Outcome valence	Level-1 feature Valence in component Evaluation
Reis (2000)	Review of the effects of relationships on development and behavior	Romantic relationships, parental relationships, friendship, coworker, neighbor	Clusters of relationships	Level-1 feature Relationship in component Relation
Bugental (2000)	Theoretical work	Attachment component, hierarchical power component, coalitional group component, reciprocity component, mating component		
Hamp & Feldweg (1997); Henrich & Hinrichs (2010, 2011)	Lexicographic database	Verbs of possession, verbs of location, verbs of emotion, social verbs, verbs of body, verbs of cognition, verbs of communication, verbs of competition, verbs of contact, verbs of natural phenomenon, verbs of creation, verbs of change, verbs of consumption, verbs of perception, stative verbs	Verbs of communication	Level-2 feature Communication of level-1 feature Act in component Activities
			Verbs of consumption	Level-2 feature Consumption of level-1 feature Act in component Activities
			Verbs of creation	Level-3 feature Mental Production of level-2 feature Production of level-1 feature Act in component Activities
			Verbs of change	Level-3 feature Physical Production of level-2 feature Production of level-1 feature Act in component Activities

Note. Theories, classifications of situations and of social situations, measurement tools, studies, and lexicographic databases are listed in chronological order in groups.

Table S4

Definitions of all Components and Features on the three different abstraction levels of the APRACE With Examples and the Underlying Literature

Component	*Level-1 Feature*	*Level-2 Feature*	*Level-3 Feature*	*Examples of Cue Words and Cue Concepts for the Coding*[1]	*Examples of Cue Words and Cue Concepts for the Coding*[1] *in the Original Study Language German*	*Examples from the Dataset*	*Examples from the Dataset in the Original Study Language German*	*Definition Inspired by*[2]
**Actor** The party from whose perspective the social interaction is assessed.	**Socio-Demographic Features** A description of the actor in terms of social and demographical components such as age and gender.	**Age** Chronological age (number of years since birth).						
		**Gender** External biological characteristics.	**Female**					
			**Male**					
	**Involvement** The degree or mode of participation in the social interaction.	**Perspective** The actor´s point of view regarding their role in the social interaction.	**Active Participant** The actor is an active participant in the social interaction.			"Playing with my kids"	"Spielen mit meinen Kindern"	
			**Indirect Participant** The actor observes one or more other people with whom they are connected through a relationship or event. The actor participates indirectly in the action on the scene, and the behavior of the actor and the interaction partner(s) is interrelated.	Watch, observe, cheer, calm, noise	Zuschauen, beobachten, anfeuern, Ruhe, Lärm	"A cyclist obviously deliberately bumped into my husband while walking"	"Beim Spaziergang hat ein Radfahrer meinen Mann offensichtlich absichtlich angerempelt"	
		**Initiation** The person or thing that gave rise to the social interaction.	**Initiation by the Actor** The actor started the social interaction.	I	Ich	"I gave someone a present"	"Habe jemandem ein Geschenk gemacht"	
			**Initiation by the Partner**	Question of	Frage von	"Wake up call from daughter"	"Weckruf der Tochter"	

Component	*Level-1 Feature*	*Level-2 Feature*	*Level-3 Feature*	*Examples of Cue Words and Cue Concepts for the Coding[1]*	*Examples of Cue Words and Cue Concepts for the Coding[1] in the Original Study Language German*	*Examples from the Dataset*	*Examples from the Dataset in the Original Study Language German*	*Definition Inspired by[2]*
			The interaction partner started the social interaction.					
			**Practical Constraint** The social interaction was caused by a circumstance that made the social interaction unavoidable.	Had to	Musste	"Successful dental treatment"	"Erfolgreiche Zahnbehandlung"	
	**Motivation** The reasons for the actor`s action.	**Biological and Physiological Needs** Survival needs for the human body (e.g., food, sleep, reproduction).		Eat, drink, restaurant, breakfast, aperitif, sex	Essen, trinken, Restaurant, Frühstück, Apéro, Sex	"Breakfast with the family”	"Gemeinsames Frühstück mit der Familie"	Maslow (1943, 1954)
		**Safety Needs** Environmental safety, without sources of danger (e.g., financial and material security, laws, social stability, health care).		Threat, provocation, police, Doctor, physiotherapy, treatment, hospital, money, finance	Bedrohung, Provokation, Polizei, Arzt, Physiotherapie, Behandlung, Krankenhaus, Geld, Finanzen	"Financial problems"	"Finanzielle Probleme"	Deci & Ryan (1997); Kasser & Ryan (1993); Maslow (1943, 1954)
		**Social Needs** To experience belonging and closeness to other people.		Date, expression of love, acceptance, rejection, cuddling, bullying, loneliness, sympathy, feeling loved, romantic, pity, familiar	Date, Liebesbekundung, Akzeptanz, Ablehnung, kuscheln, Mobbing, Einsamkeit, Sympathie, geliebt/gemocht fühlen, romantisch, Mitleid, vertraut	"Togetherness with my partner"	"Die Zweisamkeit mit meiner Lebensgefährtin"	Deci & Ryan (1997, 2000, 2002); Kasser & Ryan (1993); Maslow (1943, 1954); McClelland (1988)
		**Cognitive Needs** To gain knowledge with the aim of understanding circumstances,		Learning, tutoring, course, university, school, explaining, lecture, pupils,	Lernen, Nachhilfe, Kurs, Uni, Schule, erklären, Vorlesung, Schüler,	"Listen carefully to a lecture for four hours"	"Vier Stunden einem Vortrag aufmerksam zuhören"	Carstensen (1992); Maslow (1970)

Component	*Level-1 Feature*	*Level-2 Feature*	*Level-3 Feature*	*Examples of Cue Words and Cue Concepts for the Coding[1]*	*Examples of Cue Words and Cue Concepts for the Coding[1] in the Original Study Language German*	*Examples from the Dataset*	*Examples from the Dataset in the Original Study Language German*	*Definition Inspired by[2]*
		situations, and actions.		group work, seminar, lessons	Gruppenarbeit, Seminar, Unterricht			
		**Esteem Needs** Recognition, esteem, and respect from others.		Gratitude, appreciation, kidding, exposing, devaluation, lack of interest, paying attention, congratulations	Dankbarkeit, Wertschätzung, verarschen, blossstellen, Abwertung, Desinteresse, Aufmerksamkeit schenken, Gratulation	"My parents made fun of me"	"Meine Eltern haben sich über mich lustig gemacht"	Deci & Ryan (1997); Kasser & Ryan (1993); Maslow (1943, 1954)
		**Power Needs** To influence others' behavior.		Dominant, threatening, pointing to rules, punishment, arrogance, making demands, compulsion, upbringing, competition, victory	Dominant, drohen, Hinweis auf Regeln, Bestrafung, Arroganz, Forderungen stellen, Zwang Erziehung, Wettbewerb, Sieg	"On the job, one person disobeyed my instructions"	"Im Job hat eine Person sich meinen Anweisungen widersetzt"	McClelland (1988)
		**Self-Actualization Needs** To express one's talents and abilities.		Talent	Talent			Maslow (1943, 1954)
		**Aesthetic Needs** An active search for and appreciation of beauty and balance in design.		Pleasure, hairdresser, cosmetics	Genuss, Friseur, Coiffeur, Kosmetik	"Visit to the salon with massage"	"Besuch im Kosmetikstudie mit Massage"	Maslow (1970)
		**Achievement Needs** To reach goals, solve problems, and experience oneself as competent.		Success, competence, feeling of inferiority	Erfolg, Kompetenz, Minderwertigkeitsg efühl	"Successfully completed training"	"Erfolgreich absolviertes Training"	McClelland, Atkinson, Clark, & Lowell (1953)
		**Transcendence Needs**		Arrogance, stubbornness, superficiality,	Arroganz, Eigensinn, Oberflächlichkeit,	"When stopping briefly in the no-parking zone with	"Beim kurzen Anhalten im absoluten	Schwartz & Bardi (2001)

Component	*Level-1 Feature*	*Level-2 Feature*	*Level-3 Feature*	*Examples of Cue Words and Cue Concepts for the Coding*[1]	*Examples of Cue Words and Cue Concepts for the Coding*[1] *in the Original Study Language German*	*Examples from the Dataset*	*Examples from the Dataset in the Original Study Language German*	*Definition Inspired by*[2]
		To live generally accepted beliefs about desirable, trans-situational goals.		recklessness, self-pity, pessimism, responsibility, moodiness, impatience, avarice / greed, gratitude	Leichtsinn, Selbstmitleid, Pessimismus, Verantwortung, launisch, Ungeduld, Geiz, Gier, Dankbarkeit	my car to drop a letter, unnecessary, spiteful comments from a resident"	Halteverbot, um einen Brief einzuwerfen, unnötige, gehässige Kommentare eines Anliegers"	
		**Approach Motivation** In the current social interaction, the actor wants to approach a positive outcome.						Gable & Berkman (2008); Mehrabian & Ksionzky (1974)
		**Avoidance Motivation** In the current social interaction, the actor wants to avoid a negative outcome.		Avoid, get out of the way, push out, move, ignore	Vermeiden, aus dem Weg gehen, herausschieben, verschieben, ignorieren	"Postponing an important conversation"	"Die Herausschiebung eines wichtigen Gesprächs"	Gable & Berkman (2008); Mehrabian & Ksionzky (1974)
		**Social Support** The actor helps and supports the partner.	**Informal Support** The actor gives the partner advice, tips, suggestions, helpful information, and points out useful ways to solve the problem.	Help, support, advise, explain, tips	Helfen, unterstützen, beraten, erklären, Tipps	"Helped someone with advice"	"Jemandem geholfen durch einen Rat"	Cutrona (1986), House & Kahn (1985); Schwarzer & Schulz (2000)
			**Instrumental Support** The actor provides the partner with material help or services.	Help, support, repair, active	Helfen, unterstützen, Reparatur, tatkräftig	"I helped my neighbor move."	"Ich habe meinem Nachbarn beim Umzug geholfen"	Cutrona (1986); House & Kahn (1985); Schwarzer & Schulz (2000)
			**Emotional Support** Through his words and actions, the actor lets the partner feel empathy, understanding, and care.	Help, support worry, grief, comfort, compassion, understanding, courage, motivation	Helfen, unterstützen, Sorgen, Kummer, Trost, Mitgefühl, Verständnis, Mut, motiviert	"That someone told me worries"	"Dass mir jemand Sorgen mitgeteilt hat"	Cutrona (1986); House & Kahn (1985); Schwarzer & Schulz (2000)

Component	*Level-1 Feature*	*Level-2 Feature*	*Level-3 Feature*	*Examples of Cue Words and Cue Concepts for the Coding[1]*	*Examples of Cue Words and Cue Concepts for the Coding[1] in the Original Study Language German*	*Examples from the Dataset*	*Examples from the Dataset in the Original Study Language German*	*Definition Inspired by[2]*
		**Other** Collection specification for other motivations.						
	**Emotion** Concepts of emotional impulses such as affect, feeling, and mood.	**Basic Emotions** Essential feelings that are part of human existence and are culturally independent.	**Happiness** Pleasing feelings triggered by an event that are enjoyed and sought after.	Fun, funny, laughing, silly, joking, anticipation, rejoicing, fooling around	Spass, lustig, lachen, albern, witzeln, Vorfreude, freuen, blödeln	"Nice conversations with friends, was a lot of fun"	"Nette Gespräche mit Freunden, hat viel Spass gemacht"	Ekman (1992); Ekman & Cordaro (2011); Keltner et. al (2006), ; Zinck & Newen (2008)
			**Surprise** The reaction to a sudden unexpected event.	Spontaneous, unexpected, surprise, coincidental, suddenly, encounter	Spontan, unerwartet, Überraschung, zufällig, plötzlich, antreffen	"An unexpected call"	"Ein unerwarteter Anruf"	Graham et. al (2013); Ekman & Cordaro (2011)
			**Sadness** The reaction to the loss of a close person or a personally meaningful object.	Dejected, sorry, depressed, melancholic, sad	Niedergeschlagen, bedauern, depressiv, melancholisch, traurig	"Depressing phone call"	"Deprimierendes Telefongespräch"	Ekman & Cordaro (2011); Thompson & Crocker (2013); Zinck & Newen (2008)
			**Disgust** Repulsion by the sight, smell, or taste of something or in response to people whose actions and ideas are unappealing or offensive.	Disgusting, repugnant	Eklig, widerlich	"When shopping the shop was full of anti-social people, and they jammed up the cash register line. I find them all so disgusting, buy some alcohol shortly before closing time"	"Beim Einkaufen war der Laden voll asozialer Leute und die stauten sich dann auch noch an der Kasse, finde die alle so eklig, kurz vor Ladenschluss noch schnell	Ekman & Cordaro (2011)

Component	*Level-1 Feature*	*Level-2 Feature*	*Level-3 Feature*	*Examples of Cue Words and Cue Concepts for the Coding[1]*	*Examples of Cue Words and Cue Concepts for the Coding[1] in the Original Study Language German*	*Examples from the Dataset*	*Examples from the Dataset in the Original Study Language German*	*Definition Inspired by[2]*
							Alkohol einkaufen"	
			**Anger** The reaction to real or perceived harm (physical or psychological) or prevention from pursuing a goal.	Anger, annoying, annoying, ugly, angry	Wut, ärgerlich, nerven, hässig, sauer	"Upset about someone"	"Über jemanden aufgeregt"	Ekman & Cordaro (2011); Zinck & Newen (2008)
			**Fear** The reaction to the risk of physical or psychological injury and suffering.	Anxiety, cramped, fear	Angst, verkrampft, fürchten	"I told my husband this morning that I was afraid that I would no longer be pregnant"	"Ich habe meinem Mann heute Vormittag mitgeteilt, dass ich die Befürchtung habe, nicht mehr schwanger zu sein"	Ekman & Cordaro (2011); Thompson & Crocker (2013); Zinck & Newen (2008)
		**Non-Basic Emotions** Responses learned from the culture that are useful in that specific environment.	**Shame** A reaction to one's assumption that when their true nature becomes known, others will reject them.	Ashamed, embarrassing, disgraced	Geschämt, peinlich, blamiert	"I was embarrassed that the delivery person saw me when I had hair color in my hair"	"Es war mir peinlich, dass der Zusteller mich gesehen hat, als ich Haarfarbe in meinen Haaren hatte"	Ekman & Cordaro (2011)
			**Guilt** Regret for having violated an agreement, principle, or value.	Repentance, regret	Reue, Leid tun	"I scolded my son when he cheated. My reaction was inappropriate and exaggerated and I was sorry afterward."	"Habe meinen Sohn ausgeschimpft, als der getrödelt hat. Meine Reaktion war unangemessen und übertrieben und im Nachhinein hat es mir Leid getan"	Ekman & Cordaro (2011)
			**Envy** A person's reaction to the reward or possession of another person that they	Envious, deny	Neidisch, vergönnen	"Envy and resentment"	"Neid und Missgunst"	Ekman & Cordaro (2011)

Component	*Level-1 Feature*	*Level-2 Feature*	*Level-3 Feature*	*Examples of Cue Words and Cue Concepts for the Coding[1]*	*Examples of Cue Words and Cue Concepts for the Coding[1] in the Original Study Language German*	*Examples from the Dataset*	*Examples from the Dataset in the Original Study Language German*	*Definition Inspired by[2]*
			would like to have themselves.					
			**Jealousy** The reaction to the actions of a loved person with a third party (rival), which can be characterized by emotions such as anger, fear, or sadness.	Jealous, another man, another woman	Eifersüchtig, anderer Mann, andere Frau	"An argument with my wife about another man"	"Eine Auseinandersetzung mit meiner Ehefrau bezüglich eines anderen Mannes"	Ekman & Cordaro (2011)
			**Pride** The reaction to success, especially after completing difficult tasks or overcoming obstacles.	Proud, passed, success	Stolz, bestanden, Erfolg	"High praise for home-cooked, elaborate food"	"Hohes Lob für selbstgekochtes aufwendiges Essen"	Graham et. al (2013)
			**Doubt** Skepticism and concern about the correctness of statements made by others or whether expectations are being met.	Unsure, doubted	Unsicher, gezweifelt	"Unclear about the cash register in the supermarket. The mpties were charged questionably"	"Unklarheit beim Kassenzettel im Supermarkt. Leergut wurde fragwürdig verrechnet"	Sääksjärvi & Morel (2010)
			**Distrust** The assumption that others are not acting in one`s best interest.	Unsure, suspicious, careful	Unsicher, misstrauisch, vorsichtig	"Beggar at the front door, whom I didn't trust"	"Bettler an der Haustür, dem ich nicht getraut habe"	Rose, Peters, Shea, & Armstrong (2004)
			**Loneliness** The discrepancy between the desired and actual relationships.	Lonely, alone	Einsam, allein	"I feel alone while I'm with other people"	"Alleine fühlen während ich bei anderen Menschen bin"	Russell, Peplau, & Cutrona (1980)
			**Hope** The belief in a desirable outcome that has not yet been fulfilled.	Hope, optimistic	Hoffnung, optimistisch	"New promising contact made"	"Neuer vielversprechen-der Kontakt geknüpft"	Graham et. al (2013)
			**Melancholy/Sehnsucht** An intimate and painful craving for a person, place, or thing.	Missing them, earlier	Vermissen, damals, früher	"I received a call from people close to me telling me that I was missed. This triggered a	"Ich musste telefonisch von mir nahe stehenden Personen	O`Connor & Sussman (2014)

Component	*Level-1 Feature*	*Level-2 Feature*	*Level-3 Feature*	*Examples of Cue Words and Cue Concepts for the Coding[1]*	*Examples of Cue Words and Cue Concepts for the Coding[1] in the Original Study Language German*	*Examples from the Dataset*	*Examples from the Dataset in the Original Study Language German*	*Definition Inspired by[2]*
						similar mood in me."	telefonisch zur Kenntnis nehmen, dass ich vermisst werde. Dieses hat eine gleiche Stimmung in mir ausgelöst"	
			**Disappointment/ Frustration** The reaction to a failure to meet expectations.	Disappointed, frustrated	Enttäuscht, frustriert	"A person I once loved very much disappointed me, lied and cheated on me on several occasions"	"Eine Person, die ich einst sehr liebte, hat mich mehrfach enttäuscht, belogen und betrogen"	Marcatto & Ferrante (2008)
			**Other** Collection subspecification for other non-basic emotions					
		**Demands/ Resources** The relation between the demands on the actor and the coping resources of the actor.	**Stress** A state of tension in reaction to physical and/or mental urgency or overload.	Stress, excessive demands, too much	Stress, Überforderung, zu viel	"Deadline pressure"	"Termindruck"	Folkman & Lazarus (1984)
			**Fatigue** A state of exhaustion and lack of energy in reaction to physical and/or mental stress or overload.	Tired, limp, sluggish, exhausted, without energy	Müde, schlapp, träge, erschöpft, ohne Energie	"I was tired, exhausted, and kinked during a very personal conversation"	"Ich übermüdet, geschafft und geknickt während eines sehr persönlichen Gesprächs "	Thompson & Crocker (2013)
			**Boredome** A state of experienced monotony in reaction to repetition, under-demand, or lack of stimulation.	Boring	Langweilig	"Boring therapy session with a patient"	"Langweilige Therapiestunde mit einer Patientin"	Fisher (1993); Perkins & Hill (1985)
			**Other** Collection subspecification for other Demands/Resources ratio					

Component	*Level-1 Feature*	*Level-2 Feature*	*Level-3 Feature*	*Examples of Cue Words and Cue Concepts for the Coding*[1]	*Examples of Cue Words and Cue Concepts for the Coding*[1] *in the Original Study Language German*	*Examples from the Dataset*	*Examples from the Dataset in the Original Study Language German*	*Definition Inspired by*[2]
**Partner** The party with whom the social interaction takes place from the actor´s point of view.	**Dyadic or Group Contact** The number of partners in the social interaction.	**Dyadic Contact** The social interaction takes place between the actor and one partner.		Call, SMS conversation	Telefonat, SMS-Konversation	"Cozy evening with the girlfriend"	"Gemütlicher Abend mit der Freundin"	
		**Group Contact** The social interaction takes place between the actor and two or more partners.		Group, class, course, club	Gruppe, Klasse, Kurs, Verein	"Poker night with friends"	"Pokerabend mit Freunden"	
	**Socio-Demografic Features** Quantitative characteristics describing the partner from a social point of view.	**Age** Chronological age (the number of years since birth).	**Underage** The partner is younger than 18 years old.	Children, grandchildren, daughter, son, pubescent	Kinder, Enkel, Tochter, Sohn, pubertierend	"Arguing with my pubescent son"	"Streit mit pubertierendem Sohn"	
			**Full Age** The partner is 18 years or older.	Grandparents, parents, teachers, lecturers, bosses, work colleagues	Grosseltern, Eltern, Lehrer, Dozent, Chef, Arbeitskollege	"My mother reproached me for not contacting her for a day"	"Meine Mutter machte mir einen Vorwurf weil ich mich einen Tag lang nicht gemeldet habe"	
		**Gender** External biological characteristics.	**Female**	Girlfriend, wife	Freundin, Ehefrau	"My wife didn't agree with my suggestion for how to spend free time"	"Meine Ehefrau teilte nicht meinen Vorschlag zur Freizeitgestal-tung"	
			**Male**	Friend, colleague	Freund, Kollege	"Grilling with my uncle"	"Grillen bei meinem Onkel"	
	**Perceived Motivation** The total of the partner's motives viewed by the actor.	**Biological and Physiological Needs** To ensure the survival of the human body (e.g., need for food, sleep, reproduction).		Eat, drink, restaurant, breakfast, aperitif, sex	Essen, trinken, Restaurant, Frühstück, Apéro, Sex	"I cooked for my grown-up children and talked to them"	"Habe für meine erwachsenen Kinder gekocht und mich mit ihnen unterhalten"	
		**Safety Needs**		Threat, provocation,	Bedrohung, Provokation,	"My colleague informed me that	"Meine Kollegin hat mich darüber	Maslow (1943, 1954)

Component	*Level-1 Feature*	*Level-2 Feature*	*Level-3 Feature*	*Examples of Cue Words and Cue Concepts for the Coding[1]*	*Examples of Cue Words and Cue Concepts for the Coding[1] in the Original Study Language German*	*Examples from the Dataset*	*Examples from the Dataset in the Original Study Language German*	*Definition Inspired by[2]*
		To live in an environment without sources of danger (e.g., financial and material security, laws, social stability, health care).		police, doctor, physiotherapy, treatment, hospital, money, finance	Polizei, Arzt, Physiotherapie, Behandlung, Krankenhaus, Geld, Finanzen	we can no longer open a window in the office because of her contact lenses"	aufgeklärt, dass wir im Büro wegen ihren Kontaktlinsen nicht mehr lüften können, deshalb ist es nun total stickig"	
		**Social Needs** To experience belonging and closeness to other people.		Date, expression of love, acceptance, rejection, cuddling, bullying, loneliness, sympathy, feeling loved, romantic, pity, familiar	Date, Liebesbekundung, Akzeptanz, Ablehnung, kuscheln, Mobbing, Einsamkeit, Sympathie, geliebt/gemocht fühlen, romantisch, Mitleid, vertraut	"Affection of my grandchildren"	"Zuneigung meiner Enkel"	Deci & Ryan (1997); Kasser & Ryan (1993); Maslow (1943, 1954)
		**Cognitive Needs** To gain knowledge with the aim of understanding circumstances, situations, and actions.		Learning, tutoring, course, university, school, explaining, lecture, pupils, group work, seminar, lessons	Lernen, Nachhilfe, Kurs, Uni, Schule, erklären, Vorlesung, Schüler, Gruppenarbeit, Seminar, Unterricht	"My extra-occupational mentoring session with my mentee. She learned well and it was very relaxed."	"Meine berufsbegleitende Mentoringstunde mit meiner Mentee. Sie hat gut gelernt und es war sehr entspannt"	Deci & Ryan (1997, 2000, 2002); Kasser & Ryan (1993); Maslow (1943, 1954); McClelland (1988)
		**Esteem Needs** To receive recognition, regard, and respect from others.		Gratitude, appreciation, kidding, exposing, devaluation, lack of interest, paying attention, congratulations	Dankbarkeit, Wertschätzung, verarschen, blossstellen, Abwertung, Desinteresse, Aufmerksamkeit schenken, Gratulation	"I insulted someone"	"Ich habe jemanden beleidigt"	Carstensen (1992); Maslow (1970)

Component	***Level-1 Feature***	***Level-2 Feature***	***Level-3 Feature***	***Examples of Cue Words and Cue Concepts for the Coding[1]***	***Examples of Cue Words and Cue Concepts for the Coding[1] in the Original Study Language German***	***Examples from the Dataset***	***Examples from the Dataset in the Original Study Language German***	***Definition Inspired by[2]***
		**Power Needs** To influence others' behavior.		Dominant, threatening, pointing to rules, punishment, arrogance, making demands, compulsion, upbringing, competition, victory	Dominant, drohen, Hinweis auf Regeln, Bestrafung, Arroganz, Forderungen stellen, Zwang Erziehung, Wettbewerb, Sieg	"Strangers wanted to dictate how to park"	"Fremde wollten vorschreiben, wie man zu parken hat"	Deci & Ryan (1997); Kasser & Ryan (1993); Maslow (1943, 1954)
		**Self-Actualization Needs** To do what fits ones own talents and abilities.		Talent	Talent			McClelland (1988)
		**Aesthetic Needs** An active search for and appreciation of beauty and balance in design.		Pleasure, hairdresser, cosmetics	Genuss, Friseur, Coiffeur, Kosmetik	"Was at the hairdresser with daughter"	"War mit Tochter beim Friseur"	Maslow (1943, 1954)
		**Achievement Needs** To achieve goals, solve problems, and experience oneself as competent.		Success, competence, feelings of inferiority	Erfolg, Kompetenz, Minderwertigkeitsgefühl	"Participation in my daughter's academic success"	"Anteilnahme an akademischen Erfolgen meiner Tochter"	Maslow (1970)
		**Transcendence Needs** To live generally accepted beliefs about desirable, trans-situational goals.		Arrogance, stubbornness, superficiality, recklessness, self-pity, pessimism, responsibility, moody, impatience, avarice / greed, gratitude	Arroganz, Eigensinn, Oberflächlichkeit, Leichtsinn, Selbstmitleid, Pessimismus, Verantwortung, launisch, Ungeduld, Geiz, Gier, Dankbarkeit	"Disagreement on a moral matter with someone I actually appreciate very much in this area, but who surprised me with astonishing superficiality"	"Meinungsverschiedenheit in einer moralischen Sache mit jemandem, den ich eigentlich auf diesem Gebiet sehr schätze, der mich aber mit verblüffender Oberflächlichkeit überrascht hat"	McClelland, Atkinson, Clark, & Lowell (1953)

Component	*Level-1 Feature*	*Level-2 Feature*	*Level-3 Feature*	*Examples of Cue Words and Cue Concepts for the Coding*[1]	*Examples of Cue Words and Cue Concepts for the Coding*[1] *in the Original Study Language German*	*Examples from the Dataset*	*Examples from the Dataset in the Original Study Language German*	*Definition Inspired by*[2]
		**Approach Motivation** Wanting to approach an outcome perceived by the actor as positive.						Schwartz & Bardi (2001)
		**Avoidance Motivation** Wanting to avoid an outcome perceived by the actor as negative.		Avoid, get out of the way, push out, move, ignore	Vermeiden, aus dem Weg gehen, herausschieben, verschieben, ignorieren	"A conversation with a friend who didn't want to say what was bothering her"	"Ein nicht geführtes Gespräch mit einer Freundin, die nicht sagen wollte, was sie bedrückt"	Gable & Berkman (2008); Mehrabian & Ksionzky (1974)
		**Social Support** The help and backing of a partner.	**Informal Support** The partner gives the actor advice, tips, suggestions, helpful information and points out useful ways to solve a problem.	Help, support, advise, explain, tips	Helfen, unterstützen, beraten, erklären, Tipps	"Very good advice was given at a shopping consultation, which went down well with me"	"Bei einer Einkaufsberatung erfolgte eine sehr gute Beratung, die gut auf mich einging"	Cutrona (1986); House & Kahn (1985); Gable & Berkman (2008); Mehrabian & Ksionzky (1974); Schwarzer & Schulz (2000)
			**Instrumental Support** The partner provides the actor with material help, equipment, or services.	Help, support, repair, active	Helfen, unterstützen, Reparatur, tatkräftig	"The neighbor helped with the gardening"	"Nachbarin hat bei der Gartenarbeit geholfen"	Cutrona (1986), House & Kahn (1985); Gable & Berkman (2008); Mehrabian & Ksionzky (1974); Schwarzer & Schulz (2000)

Component	*Level-1 Feature*	*Level-2 Feature*	*Level-3 Feature*	*Examples of Cue Words and Cue Concepts for the Coding*[1]	*Examples of Cue Words and Cue Concepts for the Coding*[1] *in the Original Study Language German*	*Examples from the Dataset*	*Examples from the Dataset in the Original Study Language German*	*Definition Inspired by*[2]
			**Emotional Support** Through their words and actions, the partner lets the actor feel empathy, understanding and care.	Help, support, worry, grief, comfort, compassion, understanding, courage, motivated	Helfen, unterstützen, Sorgen, Kummer, Trost, Mitgefühl, Verständnis, Mut, motiviert	"My best friend cheered me up after bad news and invited me to spend a day together"	"Meine beste Freundin hat mich nach einer schlechten Nachricht aufgemuntert und zu einem gemeinsamen Tag eingeladen"	Cutrona (1986); House & Kahn (1985); Gable & Berkman (2008); Mehrabian & Ksionzky (1974); Schwarzer & Schulz (2000)
		**Other** Collection specification for other Motivations.						
	**Perceived Emotions** Concepts of impulses such as affections, feelings, and moods.	**Basic Emotion** Essential feelings that are part of human existence and are culturally independent.	**Happiness** Pleasing feelings triggered by an event that are enjoyed and sought after.	Fun, funny, laughing, silly, joking, anticipation, rejoicing, fooling around	Spass, lustig, lachen, albern, witzeln, Vorfreude, freuen, blödeln	"Family joy when I got home from work"	"Freude der Familie als ich von der Arbeit kam"	Ekman (1992); Ekman & Cordaro (2011); Keltner et. al (2006); Zinck & Newen (2008)
			**Surprise** The reaction to a sudden unexpected event.	Spontaneous, unexpected, surprise, coincidental, suddenly, encounter	Spontan, unerwartet, Überraschung, zufällig, plötzlich, antreffen	"Surprised someone"	"Jemanden überraschen"	Graham et. al (2013); Ekman & Cordaro (2011)
			**Sadness** The reaction to the loss of a close person or a personally meaningful object.	Dejected, sorry, depressed, melancholic, sad	Niedergeschlagen, bedauern, depressiv, melancholisch, traurig	"My life partner was very depressed about the death of his buddy"	"Mein Lebenspartner war sehr niedergeschlagen, bezüglich des Todes seines Kumpels"	Ekman & Cordaro (2011); Thompson & Crocker (2013); Zinck & Newen (2008)

Component	*Level-1 Feature*	*Level-2 Feature*	*Level-3 Feature*	*Examples of Cue Words and Cue Concepts for the Coding*[1]	*Examples of Cue Words and Cue Concepts for the Coding*[1] *in the Original Study Language German*	*Examples from the Dataset*	*Examples from the Dataset in the Original Study Language German*	*Definition Inspired by*[2]
			**Disgust** Repulsion by the sight, smell, or taste of something or repulsion in response to people whose actions and ideas are repulsive or offensive.	Disgusting	Eklig, widerlich			Ekman & Cordaro (2011)
			**Anger** The reaction to real or perceived harm (physical or psychological) or prevention from pursuing a goal.	Anger, annoying, annoying, ugly, angry	Wut, ärgerlich, nerven, hässig, sauer	"A neighbor was annoyed with my dog"	"Ein Nachbar hat sich über meinen Hund geärgert"	Ekman & Cordaro (2011); Zinck & Newen (2008)
			**Fear** The reaction to the risk of physical or psychological injury and suffering.	Fear, cramped, fear	Angst, verkrampft, fürchten	"My daughter had to go to the doctor, and she was afraid. It was not nice."	"Meine Tochter musste zum Arzt und sie hatte Angst. Das war nicht schön"	Ekman & Cordaro (2011); Thompson & Crocker (2013); Zinck & Newen (2008)
		**Non-Basic Emotions** Responses learned from the culture that are useful in that specific environment.	**Shame** A reaction to one's assumption that when their true nature becomes known, others will reject them.	Ashamed, embarrassing	Geschämt, peinlich, blamiert	"Embarrassing silence with a person"	"Peinliches Anschweigen mit einer Person"	Ekman & Cordaro (2011)
			**Guilt** Regret for having violated an agreement, principle, or value.	Repentance, Sorry	Reue, Leid tun			Ekman & Cordaro (2011)
			**Envy** A person's reaction to the reward or possession of another person that they would like to have themselves.	Envious, treat yourself	Neidisch, vergönnen	"Envy and resentment"	"Neid und Missgunst"	Ekman & Cordaro (2011)

Component	*Level-1 Feature*	*Level-2 Feature*	*Level-3 Feature*	*Examples of Cue Words and Cue Concepts for the Coding[1]*	*Examples of Cue Words and Cue Concepts for the Coding[1] in the Original Study Language German*	*Examples from the Dataset*	*Examples from the Dataset in the Original Study Language German*	*Definition Inspired by[2]*
			**Jealousy** The reaction to the actions of a loved person with a third party (rival), which can be characterized by emotions such as anger, fear, or sadness.	Jealous, another man, another woman	Eifersüchtig, anderer Mann, andere Frau	"Husband's jealousy manifestation"	"Eifersuchtsbe-kundung des Ehemannes"	Ekman & Cordaro (2011)
			**Pride** The reaction to success, especially after completing difficult tasks or overcoming obstacles.	Proud, passed, success	Stolz, bestanden, Erfolg	"Successful with a colleague"	"Mit einem Kollegen auf einen Erfolg angestossen"	Ekman & Cordaro (2011)
			**Doubt** Skepticism and concern about the correctness of statements made by others or whether expectations are being met.	Unsure, doubted	Unsicher, gezweifelt	"My grandmother had doubts about whether it would be good if I visited her with my children because my cousin told her they could make her nervous"	"Meine Grossmutter hatte Zweifel, ob es gut ist, wenn ich mit den Kindern zu ihr komme. Weil meine Cousine sich angemeldet hatte und meine Kinder Unruhe verbreiten könnten"	Graham et. al (2013)
			**Distrust** The assumption that others are not acting in one`s best interest.	Unsure, suspicious, careful	Unsicher, misstrauisch, vorsichtig	"False suspicions from colleagues"	"Falsche Verdächtigungen von Kollegen"	Sääksjärvi & Morel (2010)
			**Loneliness** The discrepancy between the desired and actual relationships.	Lonely, alone	Einsam, allein			Rose, Peters, Shea, & Armstrong (2004)
			**Hope** The belief in a desirable outcome that has not yet been fulfilled.	Hope, optimistic	Hoffnung, optimistisch	"Hope"	"Hoffnung"	Russell, Peplau, & Cutrona (1980)
			**Melancholy/Sehnsucht**	Missing then, earlier	Vermissen, damals, früher	"I received a call from people close	"Ich musste telefonisch von	Graham et. al (2013)

Component	*Level-1 Feature*	*Level-2 Feature*	*Level-3 Feature*	*Examples of Cue Words and Cue Concepts for the Coding[1]*	*Examples of Cue Words and Cue Concepts for the Coding[1] in the Original Study Language German*	*Examples from the Dataset*	*Examples from the Dataset in the Original Study Language German*	*Definition Inspired by[2]*
			An intimate and painful craving for a person, place, or thing.			to me telling me that I was missed. This triggered a similar mood in me"	mir nahe stehenden Personen telefonisch zur Kenntnis nehmen, dass ich vermisst werde. Dieses hat eine gleiche Stimmung in mir ausgelöst"	
			**Disappointment/ Frustration** The reaction to a failure to meet expectations.	Disappointed, frustrated	Enttäuscht, frustriert	"My girlfriend is so disappointed because she thinks she missed her exam"	"Meine Freundin ist so enttäuscht, weil sie glaubt ihre Prüfung versemmelt zu haben"	O`Connor & Sussman (2014)
			**Other** Collection subspecification for other non-basic emotions					Marcatto & Ferrante (2008)
		**Demands/ Resources** The relation between the demands on the partner and the coping resources of the partner as perceived by the actor.	**Stress** A state of tension in reaction to physical and/or mental urgency or overload.	Stress, excessive demands, too much	Stress, Überforderung, zu viel	"Conflict caused by general tension in the training group"	"Konflikt, ausgelöst durch allgemeine Anspannung in der Ausbildungs-gruppe"	
			**Fatigue** A state of exhaustion and lack of energy in reaction to physical and/or mental stress or overload.	Tired, limp, sluggish, exhausted, without energy	Müde, schlapp, träge, erschöpft, ohne Energie	"A visit to a customer was very annoying because he was very unmotivated and tired"	"Ein Besuch bei einem Kunden war sehr nervig, weil er sehr unmotiviert und schlapp war"	Folkman & Lazarus (1984)
			**Boredome** A state of experienced monotony in reaction to repetition, under-demand, or lack of stimulation.	Boring	Langweilig	"My grandson, who needs a lot of care, was not focused and was bored"	"Mein Enkel, der sehr viel Zuwendung braucht, stand nicht im Mittelpunkt und war gelangweilt"	Thompson & Crocker (2013)

Component	*Level-1 Feature*	*Level-2 Feature*	*Level-3 Feature*	*Examples of Cue Words and Cue Concepts for the Coding[1]*	*Examples of Cue Words and Cue Concepts for the Coding[1] in the Original Study Language German*	*Examples from the Dataset*	*Examples from the Dataset in the Original Study Language German*	*Definition Inspired by[2]*
			**Other** Collection subspecification for other Demands/Resources ratio					Fisher (1993); Perkins & Hill (1985)
**Relation** The bond between the actor and the partner from the perspective of the actor.	**Relationship** The interdependence and connection between the actor and the partner.	**Partner in Life** A person with whom a romantic relationship is being held.		Partner, wife, husband	Partner, Ehefrau, mein Mann	"Quarreling with my husband. It's always about the same things."	"Streit mit meinem Mann. Es geht immer wieder um die selben Sachen"	
		**Former Partner in Life** A person with whom a romantic relationship was being held.		Ex	Ex	"A phone conversation with my ex-wife about our children"	"Ein Telefongespräch mit meiner Ex-Frau über unsere Kinder"	
		**Relatives** People who belong to the biological or adoptive family as the actor.		Parents, brother, niece, daughter, grandmother	Eltern, Bruder, Nichte, Tochter, Grossmutter	"Phone to my brother"	"Telefonat mit meinem Bruder"	
		**Affinity** People who belong to the family of the actor through marriage.		Sister-in-law, parents-in-law	Schwägerin, Schwiegereltern	"Drinking coffee with parents-in-law"	"Kaffee trinken mit Schwiegereltern"	Swiss Civil Code (2020)
		**Friends** People with whom there is a deep emotional bond.		Friend	Freund	"Poker night with friends"	"Pokerabend mit Freunden"	Swiss Civil Code (2020)
		**Acquaintances** People with whom there is little or no deep emotional connection.		Acquaintances	Bekannte	"Visiting acquaintances in their garden"	"Bekannte in ihrem Garten besuchen"	
		**Neighbors** People who live in the (immediate) vicinity of the actor.		Neighbor	Nachbar	"Gossip with the neighbor"	"Tratsch mit dem Nachbarn"	

Component	*Level-1 Feature*	*Level-2 Feature*	*Level-3 Feature*	*Examples of Cue Words and Cue Concepts for the Coding[1]*	*Examples of Cue Words and Cue Concepts for the Coding[1] in the Original Study Language German*	*Examples from the Dataset*	*Examples from the Dataset in the Original Study Language German*	*Definition Inspired by[2]*
		**Work Contacts** People whom the actor works or volunteers with.		Boss, colleague, employee	Chef, Kollege, Mitarbeiter	"Still having trouble with a colleague at work"	"Immer noch Ärger mit einem Kollegen auf der Arbeit"	
		**School or University Contacts** People connected to the actor via school or study.		Students, lecturers, teachers	Studenten, Dozent, Lehrer	"Joint meeting for university preparation"	"Gemeinsame Besprechung für die Univorbereitung"	
		**Club Contacts** People connected to the actor via club or organization membership.		Club colleagues	Vereinskollegen	"Choir rehearsal in the men's choir"	"Chorprobe im Männerchor"	
		**Service Providers** People who provide services within an organization.		Doctor, craftsman, hairdresser, bus driver, postman	Arzt, Handwerker, Friseur, Busfahrer, Postbote	"Picking up my car from the repair shop"	"Abholung meines Autos aus der Reperatur-werkstatt"	
		**Official/Civil Servants** People who work for the government.		Civil servant	Beamter	"Unpleasant employee in the tax office"	"Unangenehme Mitarbeiterin im Finanzamt"	
		**New Contact** A person who is still largely unknown to the actor, but a relationship is in the making.		New	Neu	"New contact is difficult"	"Neuer Kontakt schwierig"	
		**Unknown Person** A person who is completely unknown and unfamiliar to the actor.		Foreign, unknown	Fremd, unbekannt	"Doing a favor for a stranger"	"Einem Fremden einen Gefallen tun"	
		**Political Contacts** People connected to the actor via		Politician, party colleague	Politiker, Parteikollege	"Election advertising distributed"	"Wahlwerbung verteilt"	

Component	*Level-1 Feature*	*Level-2 Feature*	*Level-3 Feature*	*Examples of Cue Words and Cue Concepts for the Coding[1]*	*Examples of Cue Words and Cue Concepts for the Coding[1] in the Original Study Language German*	*Examples from the Dataset*	*Examples from the Dataset in the Original Study Language German*	*Definition Inspired by[2]*
		political activity/engagement.						
		**Cohabitant** A person who lives in the same household.		Cohabitant	Mitbewohner	"Very nice pizza dinner together and evening entertainment together with the entire flat sharing community"	"Sehr schönes gemeinsames Pizzaessen und gemeinsame Abendgestaltung mit der gesamten WG"	
		**Celebrity** A person who is well known and prominent professionally or socially.		Celebrity	Berühmtheit, Prominenter	"Email from a well-known writer"	"E-Mail eines bekannten Schriftstellers"	
		**Members of a Religious Community** People who belongs to a religious community salient in the current social interaction.		Pastor, believers	Pfarrer, Gläubige	"Joint family church visit"	"Gemeinsamer familiärer Kirchgang"	
		**Other** Collection specification for other relationships						
	**Dominance Ratio** The relation between the statuses of the actor and the partner and/or the relation of perceived dominance between the actor and the partner. *Status* is the social power associated with the	**Superordinate Partner** The partner has a higher social status than the actor and/or is perceived by the actor as more dominant.		Boss, teacher, mentor, lecturer, leader, chairman, boss, professor, manager, teacher, supervisor, if something is prohibited, job, employer, supervisor	Chef, Lehrer, Mentor, Dozent, Leiter, Vorsitzender, Boss, Professor, Leitung, Lehrperson, Betreuer, wenn etwas verboten wird, Job, Arbeitgeber, Vorgesetzter	"Talking to my boss, handing over tasks, trust"	"Gespräch mit meinem Chef, Übergabe von Aufgaben, Vertrauen"	Tiedens et. al (2002); Hess, Adams, & Kleck (2005)

Component	*Level-1 Feature*	*Level-2 Feature*	*Level-3 Feature*	*Examples of Cue Words and Cue Concepts for the Coding[1]*	*Examples of Cue Words and Cue Concepts for the Coding[1] in the Original Study Language German*	*Examples from the Dataset*	*Examples from the Dataset in the Original Study Language German*	*Definition Inspired by[2]*
	actor's and partner`s roles. *Perceived dominance* is the actor's assessment of how assertive, forceful, and/or self-assured the partner is.	**Equal Partner** The partner and the actor have the same social status and/or the actor perceives the partner as equally dominant.				"Fooling around while working with colleagues and friends"	"Herumalbern auf der Arbeit mit Kollegen und Freunden"	
		**Subordinate Partner** The partner has a lower social status than the actor and/or is perceived by the actor as less dominant.		Pupils, children, mentees, employees, apprentices, trainees, learners, students, employees, subordinates, forbid something, tutoring, grandchildren	Schüler, Kinder, Mentee, Mitarbeiter, Lehrling, Auszubildende, Lernende, Studierende, Angestellte, Untergebene, etwas verbieten, Nachhilfe, Enkel, Grosskind	"Excursion with the assistant"	"Ausflug mit der Assistentin"	
	**Closeness** Interconnection and oneness with the partner as perceived by the actor, which involves interconnected identities.	**Closeness in General** The actor`s overall feeling of interconnectedness with the partner.	**Close** The actor generally feels close to the partner.	Closely connected, close, familiar	Eng verbunden, nah, vertraut, nahestehend	"A person closely related to me expressed and behaved very negatively"	"Eine mir eng verbundene Person hat sich sehr negativ geäussert und verhalten"	Cialdini, Brown, Lewis, Luce & Neuberg (1997)
			**Not Close** The actor generally does not feel close to the partner.			"There was a friction with a stranger while shopping"	"Es gab eine Reiberei mit einer fremden Person beim Einkaufen"	
		**Closeness in the Situation** The actor`s feeling of interconnectedness with the partner in the specific social interaction.	**Close** In the specific situation, the actor feels close to the partner.	Trust, distance	Vertrauen, Distanz	"To feel the connection with the partner through conversations and physical attachment"	"Die Verbundenheit mit der Partnerin durch Gespräche und körperliche Anhänglichkeit zu spüren"	Cialdini, Brown, Lewis, Luce & Neuberg (1997)
			**Not Close** In the specific situation, the actor does not feel close to the partner.			"Connected relatively shortly as telephone contact"	"Mit telefonischem Kontakt relativ kurz angebunden"	

Component	*Level-1 Feature*	*Level-2 Feature*	*Level-3 Feature*	*Examples of Cue Words and Cue Concepts for the Coding[1]*	*Examples of Cue Words and Cue Concepts for the Coding[1] in the Original Study Language German*	*Examples from the Dataset*	*Examples from the Dataset in the Original Study Language German*	*Definition Inspired by[2]*
**Activities** Interdependent behaviors between the actor and the partner in the social interaction from the perspective of the actor.	**Act** What the actor and the partner do in the social interaction.	**Communication** The focus of the interaction between the actor and the partner conveyed by messages in the social interaction.		Conversation, talking, telephone, writing, greeting/saying goodbye, arguing, discussing	Gespräch, reden, Telefon, schreiben, begrüssen, verabschieden, streiten, diskutieren	"Dialogue with my mother "	"Gespräch mit meiner Mutter"	Hamp & Feldweg (1997); Henrich & Hinrichs (2010, 2011)
		**Consumption** Procurement of necessities and goods and their use and intake during the social interaction.	**Physical** In social interaction, physical or concrete/objective consumption takes place.	Eating, shopping, health, beauty, wellness, ordering, delivered, being able to pick up, craftsmen, train, bus	Essen, einkaufen, Gesundheit, Schönheit, Wellness, bestellen, geliefert, abholen können, Handwerker, Zug, Bus	"Long-sought wood has finally been bought"	"Lang gesuchtes Holz endlich gekauft"	
			**Mental** In social interaction, consumption takes place on an intellectual and/or mental level.	Participate in an event (e.g., concert, theater), media consumption (e.g., TV, cinema), look at photos, expand knowledge as part of an event (e.g., lecture), guided tours, celebrations	An einer Veranstaltung teilnehmen (z.B. Konzert, Theater), Medienkonsum (z.B. TV, Kino), Fotos anschauen, Wissenserweiterung im Rahmen einer Veranstaltung (z.B. Vorlesung), Führung, Feste feiern	"Watch the film"	"Den Film schauen"	
		**Production** Something new created, generated, or produced during the social interaction.	**Physical** The production output is perceptible with all senses (i.e., tangible, visible).	Gardening, cooking, or baking (special, not everyday cooking), creating, making, mucking out, setting up, building	Gartenarbeit, kochen oder backen (sofern es nicht das alltägliche Kochen ist, sondern etwas Spezielles gekocht wird), erstellen, machen, ausmisten, einrichten, Aufbau	"I visited a dear friend at home, and we cooked lunch together"	"Ich habe eine liebe Freundin zu Hause besucht und wir haben zusammen Mittag gekocht"	

Component	*Level-1 Feature*	*Level-2 Feature*	*Level-3 Feature*	*Examples of Cue Words and Cue Concepts for the Coding[1]*	*Examples of Cue Words and Cue Concepts for the Coding[1] in the Original Study Language German*	*Examples from the Dataset*	*Examples from the Dataset in the Original Study Language German*	*Definition Inspired by[2]*
			**Mental** The production output is abstract and perceptible on an intellectual level.	Learning, group work, teaching, teaching, planning, organization, line discussion, meeting, consultation, brainstorming, negotiation, singing and playing music	Lernen, Gruppenarbeiten, lehren, beibringen, planen, Organisation, Liniengespräch, Sitzung, Absprache, Brainstorming, Verhandlung, singen und musizieren	"Planning an upcoming major event together"	"Gemeinsame Planung eines bevorstehenden grossen Ereignisses"	
		**Physical Activity**	**Mild Intensity** From the actor's perspective, physical activity is of mild intensity.	Lounging, hanging out, relaxation, wellness, massage, bed, cuddling	Gammeln, hängen, Erholung, Wellness, Massage, Bett, kuscheln	"Cuddling with the children"	"Kuscheln mit den Kindern"	
			**Medium Intensity** From the actor's perspective, physical activity is of medium intensity.	Walk, excursion/travel, bike ride	Spaziergang, Ausflug, Reisen, Velofahrt	"I went for a walk with my family in the great weather"	"Ich habe einen Spaziergang mit meiner Familie gemacht bei tollem Wetter"	
			**High Intensity** From the actor's perspective, physical activity is of high intensity.	Sports, tennis, fitness, walking, walking, hiking, climbing/bouldering, dancing	Sport, Tennis, Fitness, Walking, Walken, Wandern, Klettern, Bouldern, Tanzen	"Bike tour with my wife"	"Radtour mit meiner Frau"	
		**Play** A game or any activity without a conscious purpose, done for pleasure, relaxation, or the joy of the activity itself.		Play	Spielen	"Water battle with my grandchildren"	"Wasserschlacht mit meinen Enkeln"	
		**Mundanity, Routines**		Housework, cooking (everyday, not	Haushaltsarbeiten, kochen (sofern damit alltägliches	"Put the 8-year-old daughter to	"Das Zubettbringen der	

Component	*Level-1 Feature*	*Level-2 Feature*	*Level-3 Feature*	*Examples of Cue Words and Cue Concepts for the Coding[1]*	*Examples of Cue Words and Cue Concepts for the Coding[1] in the Original Study Language German*	*Examples from the Dataset*	*Examples from the Dataset in the Original Study Language German*	*Definition Inspired by[2]*
		Activities that are part of the daily routine at home or domestic needs that serve to maintain a household.		special cooking), repairs, getting up, going to bed, coming home, everyday life, washing, clearing, cleaning, receiving deliveries, daily/weekly planning at home	kochen gemeint ist), Reparatur, Aufstehen, zu Bett gehen, nach Hause kommen, Alltag, (ab)wasch, abräumen, putzen, Lieferungen empfangen, alltägliche/wöchentliche Planungen zu Hause	bed and read aloud"	8-jährigen Tochter mit lesen"	
		**Other** Collection specification for other Acts						
	**Interaction Mode** The form of communication used to interact with each other.	**Oral** The actor and the partner have verbal conversation.	**Face-to-Face** The actor and the partner, which are physically present, have a conversation.	Visit, meeting, seminar	Besuch, Treffen, Sitzung, Seminar	"Conversation in the cafeteria"	"Gespräch in der Mensa"	
			**Media** The actor and the partner have a direct conversation via a medium (e.g., phone, video call).	Call, Skype	Telefonat, Skype	"Long phone call with a good friend”	"Langes Telefonat mit gutem Freund"	
			**Voice message** The actor and the partner communicate at different times via a medium.	Voice message	Sprachnachricht			
		**Written** The actor and the partner communicate using written messages.	**Handwritten** The actor and the partner communicate using handwritten messages (e.g., letter).	Letter	Brief	"I received a letter that made me sad"	"Habe einen Brief erhalten, der mich traurig machte"	
			**Media** The actor and the partner communicate using written messages via an	Email, Whatsapp, SMS	E-Mail, Whatsapp, SMS	"Annoying emails"	"Nervige E-Mails"	

Component	*Level-1 Feature*	*Level-2 Feature*	*Level-3 Feature*	*Examples of Cue Words and Cue Concepts for the Coding[1]*	*Examples of Cue Words and Cue Concepts for the Coding[1] in the Original Study Language German*	*Examples from the Dataset*	*Examples from the Dataset in the Original Study Language German*	*Definition Inspired by[2]*
			electronic medium (e.g., email, chat).					
		**Paraverbal** Communication by means of voice characteristics and speaking behavior, whereby the entire spectrum of the voice is included (e.g., volume of the voice).	**Appropriate Volume** The actor and the partner communicated with each other at a comfortable volume.					
			**Inappropriate Volume** The actor and the partner communicated with each other too loudly.	Scream, loud	Schreien, laut	"I was yelled at by my sister"	"Ich wurde von meiner Schwester angeschrien"	
		**Say Nothing** No words are exchanged as part of a social interaction.	**Both** Both the actor and the partner remain silent during the social interaction.	Quiet, silent, say nothing, without words	Ruhig, schweigen, nichts sagen, ohne Worte, still	"I need rest during my smoking break and like to be alone. Unfortunately someone came in from the same floor today, but didn't say anything, so I tensed up instead of relaxing."	"Bei meiner Raucherpause brauche ich Ruhe und bin gern alleine, leider kam heute noch jemand aus derselben Etage hinzu, hat aber nichts geredet, dabei verspanne ich total statt zu entspannen"	
			**Actor** The actor remains silent during the social interaction.	Quiet, silent, say nothing, without words	Ruhig, schweigen, nichts sagen, ohne Worte, still			
			**Partner** The partner remains silent during the social interaction.	Quiet, silent, say nothing, without words	Ruhig, schweigen, nichts sagen, ohne Worte, still			
		**Nonverbal** The actor and the partner communicate using mimicry or gestures.	**Mimicry** The actor and the partner communicate using facial expressions (e.g., smile).	Change of look, smile, nod	Blickwechsel, lächeln, nicken	"During soccer training, I constantly played the ball poorly. It was looked at	"Beim Fussballtraining andauernd den Ball verschossen im Spiel. Wurde von den	

Component	*Level-1 Feature*	*Level-2 Feature*	*Level-3 Feature*	*Examples of Cue Words and Cue Concepts for the Coding[1]*	*Examples of Cue Words and Cue Concepts for the Coding[1] in the Original Study Language German*	*Examples from the Dataset*	*Examples from the Dataset in the Original Study Language German*	*Definition Inspired by[2]*
						badly by the other players."	Mitspielern böse angeschaut"	
			**Gesture**<br>The actor and the partner communicate using finger and hand movements (e.g., wave).	hand	Hand	"Car driver gave me the finger"	"Autofahrer zeigt Stinkefinger"	
			**Entire Body**<br>The actor and the partner communicate using the entire body (e.g., dance).	To dance	Tanzen	"Rain dance with my wife"	"Regentanz mit meiner Frau"	
	**Physical Contact**<br>The actor and the partner communicate using physical contact.	**With Physical Contact**<br>During the social interaction, the actor and the partner have physical contact.		Cuddling, sex, bed, dancing, holding hands, touching, massage, hairdressing, cosmetics	Kuscheln, Sex, Bett, tanzen, Händchen halten, berühren, Massage, Friseur, Kosmetik	"Kisses from RN"	"Küsse von RN"	
		**Without Physical Contact**<br>During the social interaction, the actor and the partner have no physical contact.						
	**Quality**<br>Significance of the social interaction for the actor.	**Superficial**<br>The social interaction is incidental and/or in superficial.		Small talk, irrelevant, fooling around, silly, exchange, scheduling, weather	Smalltalk, belanglos, blödeln, albern, Wortwechsel, Terminplanung, Wetter	"Smalltalk on the toilet; I don't really know the person, find her likable and have the impression that she sees it the same way"	"Ein Smalltalk auf der Toilette, weil ich die Person nicht wirklich kenne, sie sympathisch finde und den Eindruck habe, dass sie das genauso sieht"	
		**Intimate**<br>A deepening of the content can be observed in social interaction.		Worries, help, discussion, lecture, long conversation, doctor visits,	Sorgen, Hilfe, Diskussion, Vorlesung, langes Gespräch, Arztbesuche,	"Stimulating conversation"	"Anregendes Gespräch"	

Component	*Level-1 Feature*	*Level-2 Feature*	*Level-3 Feature*	*Examples of Cue Words and Cue Concepts for the Coding[1]*	*Examples of Cue Words and Cue Concepts for the Coding[1] in the Original Study Language German*	*Examples from the Dataset*	*Examples from the Dataset in the Original Study Language German*	*Definition Inspired by[2]*
				thanksgiving, meeting	Danksagung, Sitzung			
	**Anticipation of the Interaction** The expectancy of the social interaction.	**Habit** The social interaction takes place regularly, repeatedly, and in a constant form and/or procedure.		Regular, ritual, traditional, usual, repeated, again, as always, the ending "-ing" when specifying the time of day (e.g., in the morning)	Regelmässig, rituell, traditionell, gewohnt, wiederholt, erneut, wie immer, die Endung"-dlich" bei Angabe der Tageszeit (z.B. morgendlich)	"Weekly yoga class"	"Wöchentliche Yogastunde"	
		**Scheduled** The social interaction was planned.		Planned, invitation	Geplant, Einladung	"Court Hearing"	"Gerichtstermin"	
		**Unexpected by the Actor and the Partner** From the actor`s and partner`s perspectives, the social interaction takes place spontaneously or accidentally.		Spontaneous, unexpected, coincidental, suddenly, encounter	Spontan, unerwartet, zufällig, plötzlich, antreffen	"I thought I had to have lunch alone today, but a few friends spontaneously accompanied me"	"Ich dachte, ich müsste heute allein zu Mittag essen, aber spontan haben mich ein paar Freunde begleitet"	
		**Unexpected by the Actor** From the actor`s perspective, the social interaction takes place spontaneously or accidentally.		Spontaneous, unexpected, surprise	Spontan, unerwartet, Überraschung	"Girlfriend made me a surprise"	"Freundin hat mir eine Überraschung gemacht"	
		**Unexpected by the Partner** From the partner`s perspective, the social interaction takes place		Spontaneous, unexpected, surprise	Spontan, unerwartet, Überraschung	"Given a birthday gift"	"Geburtstags-geschenk überreicht"	

Component	*Level-1 Feature*	*Level-2 Feature*	*Level-3 Feature*	*Examples of Cue Words and Cue Concepts for the Coding[1]*	*Examples of Cue Words and Cue Concepts for the Coding[1] in the Original Study Language German*	*Examples from the Dataset*	*Examples from the Dataset in the Original Study Language German*	*Definition Inspired by[2]*
		spontaneously or accidentally.						
	**Anticipation of the Topic/Content** The expectancy of what the social interaction will be about.	**Expected by the Actor and the Partner** The topic/content of the social interaction is known in advance by the actor and the partner.		Expected to be right, confirmed	Erwartet, Recht haben, bestätigt			
		**Unexpected by the Actor and the Partner** The topic/content of the social interaction is not known in advance by the actor and the partner.		Unexpected, unexpected, (not) working, unsuccessful, relieved	Unerwartet, unverhofft, (nicht) funktionieren, erfolglos, erleichtert			
		**Unexpected by the Actor** The topic/content of the social interaction is not known in advance by the actor.		Says, asks, tells, reports	Sagt, fragt, erzählt, berichtet	"Unexpectedly high costs for the repair of the craftsman"	"Unerwartet hohe Kosten für die Reparatur des Handwerkers"	
		**Unexpected by the Partner** The topic/content of the social interaction is not known in advance by the partner.		Says, asks, tells, reports	Sagt, fragt, erzählt, berichtet	"Son was a bit rude and was not happy about his presents"	"Sohn war etwas unfreundlich und hat sich nicht über seine Geschenke gefreut"	
**Context** External characteristi	**Location**	**At the Actor`s Home**		Receive a visit, garden, home, home office	Besuch empfangen, Garten, zu Hause, Homeoffice	"Friends were visiting"	"Freunde waren zu Besuch"	

Component	*Level-1 Feature*	*Level-2 Feature*	*Level-3 Feature*	*Examples of Cue Words and Cue Concepts for the Coding*[1]	*Examples of Cue Words and Cue Concepts for the Coding*[1] *in the Original Study Language German*	*Examples from the Dataset*	*Examples from the Dataset in the Original Study Language German*	*Definition Inspired by*[2]
cs of the situation in which a social action is embedded from the perspective of the actor.	The place where the social interaction takes place.	**At the Partner`s Home**		Make a visit	Besuch machen	"To unknown persons in the apartment"	"Zu Unbekanntem in die Wohnung"	
		**Work Place**		Interactions with boss and work colleagues, treatment of patients	Interaktionen mit Chef und Arbeitskollegen, Behandlung von Patienten	"Help in the office"	"Hilfe im Büro"	
		**Healthcare Facility/Institution**		Physiotherapy, psychotherapy, hospital, treatment	Physiotherapie, Psychotherapie, Krankenhaus, Behandlung	"Visited my sister in the hospital"	"Besuchte meine Schwester im Krankenhaus"	
		**Shopping Location**		Supermarket, cash register, market	Supermarkt, Kasse, Markt	"Conversation at the supermarket checkout"	"Gespräch an der Supermarktkasse"	
		**Education Institution/Library**		University, school, library, seminar, class	Uni, Schule, Bibliothek, Seminar, Klasse	"Met a friend in the library, had a nice chat and had a coffee"	"Habe in der Bibliothek eine Bekannte getroffen, haben uns nett unterhalten und haben noch einen Kaffee getrunken"	
		**Cultural Place/Religious Site**		Church	Kirche	"Have been with many people during and after a worship service"	"Bei und nach einem Festgottesdienst mit vielen Menschen zusammenge-wesen"	
		**Government Office**		Court, office	Gericht, Amt	"Court Hearing"	"Gerichtstermin"	
		**Sports Facility**		Gym, swimming pool, sports field	Fitnessstudio, Schwimmbad, Sportplatz	"Been to the sports studio again"	"Wieder mal im Sportstudio gewesen"	
		**Public Transport/Traffic**		Train stations, drive home, park, playground, train, bus, traffic	Bahnhöfe, Heimfahrt, Park, Spielplatz, Zug, Bus, Verkehr	"Stress with bus drivers"	"Stress mit Busfahrer"	

Component	*Level-1 Feature*	*Level-2 Feature*	*Level-3 Feature*	*Examples of Cue Words and Cue Concepts for the Coding[1]*	*Examples of Cue Words and Cue Concepts for the Coding[1] in the Original Study Language German*	*Examples from the Dataset*	*Examples from the Dataset in the Original Study Language German*	*Definition Inspired by[2]*
		**Nature**		Hiking, lake, forest	Wandern, See, Wald	"Sunbathing at the lake with a friend"	"Sonnen am See mit einer Freundin"	
		**Culinary Establishment/Hotel**		Restaurant, hotel	Restaurant, Hotel	"In a bar with best friends"	"In einer Bar mit den besten Freunden"	
		**Other** Collection specification for other Locations						
	**Event** A time-limited happening with a thematic/content-related purpose or intended use.	**Visit** The social interaction takes place in the context of the actor's temporary stay at the partner's current place of residence (partner's household, home accommodation, camp accommodation, hotel room, hospital room). The actor is a guest of the partner.		Visit	Besuch	"Visitors were way too early"	"Besuch war viel zu früh"	
		**Leisure Event** The actor and partner take part in an event in their leisure time or for pleasure.		Concert, cinema, festival, theater, street festival, escape room	Konzert, Kino, Festival, Theater, Strassenfest, Escaperoom	"Saw the theater performance”	"Theateraufführung gesehen"	
		**Celebration** The actor and partner celebrate together at a party. In addition to the normative		Birthday, wedding, anniversary	Geburtstag, Hochzeit, Jubiläum	"My sister's wedding-eve party"	"Junggesellinnen-abschied meiner Schwester"	

Component	*Level-1 Feature*	*Level-2 Feature*	*Level-3 Feature*	*Examples of Cue Words and Cue Concepts for the Coding*[1]	*Examples of Cue Words and Cue Concepts for the Coding*[1] *in the Original Study Language German*	*Examples from the Dataset*	*Examples from the Dataset in the Original Study Language German*	*Definition Inspired by*[2]
		celebrations (e.g., birthdays), this also includes all other organized celebrations.						
		**Event of Knowledge Transfer** The actor and partner take part in organized events to expand knowledge. This includes courses, seminars, training courses, conferences, trade fairs of all contents.		Course, seminar, school, lecture	Kurs, Seminar, Schule, Vortrag	"We had a seminar on stress in the company"	"Wir hatten in der Firma ein Seminar zu Stress"	
		**Meeting** The actor and the partner take part in a meeting with specifically predefined content as part of an organization.		Meeting	Sitzung, Besprechung	"A meeting in the company"	"Eine Besprechung in der Firma"	
		**Convention** The actor and the partner take part in a gathering of a large group that has come together for a specific purpose and/or common goal.		Assembly	Versammlung	"Reception at the General Assembly with 35 members, nice conversations"	"Empfang zur Generalversammlung mit 35 Mitgliedern, nette Gespräche"	
		**Religious Event** The actor and partner take part in an event with a religious		Worship, confirmation, wedding	Gottesdienst, Konfirmation, Hochzeit	"Bible study - was very nice tonight, lots of inspiration"	"Bibelkreis - war sehr schön heute Abend, viele Inspirationen"	

Component	*Level-1 Feature*	*Level-2 Feature*	*Level-3 Feature*	*Examples of Cue Words and Cue Concepts for the Coding[1]*	*Examples of Cue Words and Cue Concepts for the Coding[1] in the Original Study Language German*	*Examples from the Dataset*	*Examples from the Dataset in the Original Study Language German*	*Definition Inspired by[2]*
		background and a religious mindset.						
		**Club Event** The actor and the partner jointly pursue a certain common interest within the context of an official association.		Club, training, rehearsal	Verein, Training, Probe	"Visited a new volleyball group that was a lot of fun"	"Eine neue Volleyballgruppe besucht, bei der es viel Spass gemacht hat"	
		**Day Trip/Journey** The actor and the partner are together on the way to a certain place with the aim of relaxing or experiencing new things.		Vacation, excursion	Urlaub, Ausflug	"Excursion with wife to a port city"	"Ausflug mit Gattin zu einer Hafenstadt"	
		**Services** The actor uses services of an organization and receives services.	**Shipping Service** The actor sends or receives objects.	Parcel, post, messenger, delivery	Paket, Post, Bote, Lieferung	"I sent a parcel to my daughter, who was married in Australia"	"An meine in Australien verheiratete Tochter ein Päckchen geschickt"	
			**Public Transport** The actor uses public transportation.	Train, tram, bus	Zug, Tram, Bus	"Talking to someone on the train"	"Gespräch in der Bahn mit einer Mitreisenden"	
			**Craftsperson** The actor uses the services of a craftsperson.	Craftsperson, craftsmen	Handwerker	"Customer service from a repair company"	"Kundendienst einer Reparaturfirma"	
			**Health** The actor seeks out a person who is knowledgeable about health issues and/or works in a related organization and can help.	Doctor, physiotherapy, psychotherapy, hospital	Arzt, Physiotherapie, Psychotherapie, Krankenhaus	"Moments of well-being during physiotherapy"	"Bei der Physiotherapie Wohlfühlmo-mente"	
			**Beauty**	Hairdresser, cosmetics	Friseur, Coiffeur, Kosmetik	"Manicure appointment with	"Maniküretermin mit	

Component	*Level-1 Feature*	*Level-2 Feature*	*Level-3 Feature*	*Examples of Cue Words and Cue Concepts for the Coding[1]*	*Examples of Cue Words and Cue Concepts for the Coding[1] in the Original Study Language German*	*Examples from the Dataset*	*Examples from the Dataset in the Original Study Language German*	*Definition Inspired by[2]*
			The actor seeks out a person who is knowledgeable about physical appearance issues and/or works in a related organization and can help.			subsequent cosmetic treatment"	anschliessender Kosmetikbehand-lung"	
			**Other** Collection subspecification for other Services					
		**Other** Collection specification for other events						
	**Setting** The space in which the social interaction takes place.	**Private** The actor and the partner interact in private space. Rooms that are owned by private individuals are referred to as private space (e.g., own house/apartment, own car, own garden).		House, apartment, cooking, TV, household chores, car	Haus, Wohnung, kochen, TV, Haushaltsarbeiten, Auto	"Husband turns the TV up to not hear what I'm saying!"	"Ehemann schaltet den Fernseher lauter, um nicht zu hören was ich sage!"	
		**Public** The actor and the partner interact in public space, ones freely accessible to all and maintained by an organization.		Supermarket, university, street, government agency	Supermarkt, Universität, Strasse, Behörde	"Going to the cinema with a friend"	"Kinobesuch mit einem Freund"	
		**Virtual** The actor is in an interactive online platform where the		Online, forum, game	Online, Forum, Game	"Won online poker“	"Beim Onlinepoker gewonnen"	

Component	*Level-1 Feature*	*Level-2 Feature*	*Level-3 Feature*	*Examples of Cue Words and Cue Concepts for the Coding[1]*	*Examples of Cue Words and Cue Concepts for the Coding[1] in the Original Study Language German*	*Examples from the Dataset*	*Examples from the Dataset in the Original Study Language German*	*Definition Inspired by[2]*
		social interaction takes place.						
	**Surroundings** The environment in which the social interaction takes place.	**Indoors/Inside** The social interaction takes place inside a room or in a vehicle.		House, apartment, car	Haus, Wohnung, Auto	"A visit to the opera"	"Ein Opernbesuch"	
		**Outdoors/Outside** The social interaction takes place outdoors. This includes all types of shelters.		Walk, forest, garden	Spaziergang, Wald, Garten	"Failure in the garden"	"Misserfolg im Garten"	
	**Frame** The social context in which the interaction takes place.	**Private** The actor and the partner interact in a private context.	**Informal** In the private frame, the social interaction takes place freely and without a formal obligation.	Going to the cinema, interactions with family members (e.g., wife, uncle, son) and friends	Kinobesuch, Interaktionen mit Familienangehö-rigen (z.B. Ehefrau, Onkel, Sohn) und Freunden	"Long conversation with my grandma"	"Längeres Gespräch mit meiner Oma"	
			**Formal** In the private frame, the social interaction takes place in the context of a (official) function or task to be carried out.	Meeting with the parents, education	Elterngespräch, Erziehung	"Parent talk at school was constructive"	"Elterngespräch war konstruktiv"	
		**Professional** The actor and the partner interact in a professional or volunteer context.	**Informal** In the professional frame, the social interaction takes place freely and without a formal obligation.	Interactions in the workplace, eat, drink, trip	Interaktionen am Arbeitsplatz, essen, trinken, Ausflug	"A colleague wanted to ask me about my private life"	"Ein Kollege wollte mich über mein Privatleben aushorchen"	
			**Formal** In the professional frame, the social interaction takes place in the context of a (official) function or a task to be carried out.	Boss, meeting, customer, student, work, job	Chef, Besprechung, Kunde, Schüler, Arbeit, Job	"Appreciation from the boss"	"Wertschätzung vom Chef"	

Component	*Level-1 Feature*	*Level-2 Feature*	*Level-3 Feature*	*Examples of Cue Words and Cue Concepts for the Coding*[1]	*Examples of Cue Words and Cue Concepts for the Coding*[1] *in the Original Study Language German*	*Examples from the Dataset*	*Examples from the Dataset in the Original Study Language German*	*Definition Inspired by*[2]
		**Political** The actor and the partner interact in a political context.	**Informal** In the political frame, the social interaction takes place freely and without a formal obligation.	Political conversation, demonstration	Politisch Gespräch, Demonstration	"While shopping I talked about the elections"	"Beim Einkaufen habe ich mich über die Wahlen unterhalten"	
			**Formal** In the political frame, the social interaction takes place in the context of a (official) unction or a task to be carried out.	Election, party	Wahl, Partei	"Meeting with fellow campaigners of a citizens' initiative"	"Zusammen-treffen mit Mitstreitern einer Bürgerinitiative"	
	**Day Time** A certain time of a day.	**In the Morning** The social interaction takes place in the morning.		In the morning, breakfast, get up	Morgens, Frühstück, aufstehen	"Woken up by my daughter"	"Aufgeweckt von meiner Tochter"	
		**Noon** The social interaction takes place at noon.		Lunch, brunch	Mittagessen, Brunch	"Unfriendly service in the restaurant at lunch"	"Unfreundliche Bedienung im Restaurant beim Mittagessen"	
		**Afternoon** The social interaction takes place in the afternoon.		Afternoon	Nachmittag	"Afternoon coffee with a friend"	"Nachmittags Kaffee trinken mit einer Freundin"	
		**In the Evening** The social interaction takes place in the evening.		Evening	Abend, Ausgang	"Series evening with my roommate"	"Serienabend mit meinem Mitbewohner"	
		**At Night** The social interaction takes place in the night.		Night, sleep, bed	Nacht, Schlaf, Bett	"Hours together with my partner on a mild summer night"	"Gemeinsame Stunden mit meinem Partner in einer lauen Sommernacht"	
	**Time Since Last Interaction** The subjective feeling by the actor	**A Short Time Ago** The actor feels that the last social interaction with the						

Component	*Level-1 Feature*	*Level-2 Feature*	*Level-3 Feature*	*Examples of Cue Words and Cue Concepts for the Coding*[1]	*Examples of Cue Words and Cue Concepts for the Coding*[1] *in the Original Study Language German*	*Examples from the Dataset*	*Examples from the Dataset in the Original Study Language German*	*Definition Inspired by*[2]
	of how long ago the last interaction with the current interaction partner was.	current interaction partner had taken place a short time ago.						
		**A Long Time Ago** The actor feels that the last social interaction with the current interaction partner had taken place a long time ago.		Long ago, in the past, former, old, after months, for weeks	Lang, früher, ehemalig, alt, nach Monaten, wochenlang	"I met up with good friends after months"	"Habe mich mit guten Freunden nach Monaten wieder getroffen"	
	**Duration** How much time the social interaction took.	**Short** The social interaction lasted about 15 minutes or less.		In short, compliments, greetings, SMS, congratulations, doctor's consultation	Kurz, Kompliment, Begrüssung, SMS, Gratulation, Arztkonsultation	"Had a short but good conversation"	"Hatte ein kurzes, aber gutes Gespräch"	
		**Long** The social interaction lasted more than 15 minutes.		Long, eating, events (e.g., concert), excursion, sporting activities, lecture	Lang, essen, Veranstaltungen (z.B. Konzert), Ausflug, sportliche Aktivitäten, Vorlesung	"Hike up a mountain with two friends"	"Wanderung auf einen Berg mit zwei Freunden"	
	**Time Focus** The temporal focus of the content of social interaction.	**Past** The social interaction is past-oriented.		Reminding, interaction about the deceased, looking at photos, retrospective, reflection	Erinnern, Interaktion über verstorbene Person, Fotos anschauen, Retrospektive, Reflexion	"Received a nice souvenir photo"	"Schönes Erinnerungsfoto erhalten"	
		**Present** The social interaction is present-oriented.						
		**Future** The social interaction is future-oriented.		Planning, making appointments, making new contacts, anticipation,	Planen, Terminvereinba-rung, neue Kontakte knüpfen, Vorfreude, drohen,	"Joint construction planning continued"	"Gemeinsame Bauplanung weitergeführt"	

Component	*Level-1 Feature*	*Level-2 Feature*	*Level-3 Feature*	*Examples of Cue Words and Cue Concepts for the Coding[1]*	*Examples of Cue Words and Cue Concepts for the Coding[1] in the Original Study Language German*	*Examples from the Dataset*	*Examples from the Dataset in the Original Study Language German*	*Definition Inspired by[2]*
				threatening, postponing, making demands, political activities, education	herausschieben, Forderungen stellen, politische Aktivitäten, Erziehung			
	**Course** The structural process of the social interaction.	**Continuity** Specification of the course component and inclusion of the temporal component.	**Continuously** The social interaction took place continuously, without interruption.					
			**Intermittent** Social interaction has been disrupted or interrupted by third parties or external circumstances.	Interruption, disturbed, pause	Unterbruch, gestört, Pause			
			**Premature Start** Two interaction parties are scheduled to interact at an official (fixed time of the event), unofficial (no fixed time, but socially accepted / habitual duration of an event) or personally determined time. The social interaction begins before this agreed or expected time.	Missed early	Früh, verpasst			
			**Delayed Start** Two interaction parties are scheduled to interact at an official (fixed time of the event), unofficial (no fixed time, but socially accepted / habitual duration of an event) or personally determined time. One interaction party is late	Wait, late	Warten, spät	"Waiting for a doctor for an hour despite having an appointment"	"Eine Stunde Warten auf einen Arzt und dies trotz Termins"	

Component	*Level-1 Feature*	*Level-2 Feature*	*Level-3 Feature*	*Examples of Cue Words and Cue Concepts for the Coding[1]*	*Examples of Cue Words and Cue Concepts for the Coding[1] in the Original Study Language German*	*Examples from the Dataset*	*Examples from the Dataset in the Original Study Language German*	*Definition Inspired by[2]*
			and makes the other interaction party wait.					
			**Premature End** Two interaction parties are scheduled to interact at an official (fixed time of the event), unofficial (no fixed time, but socially accepted / habitual duration of an event) or personally determined time. One interaction party ends the social interaction before the agreed or expected end of the social interaction.	Early, termination	Früh, Abbruch	"Early closing time"	"Früher Feierabend"	
			**Delayed End** Two interaction parties are scheduled to interact at an official (fixed time of the event), unofficial (no fixed time, but socially accepted / habitual duration of an event) or personally determined time. One interaction party ends the social interaction after the agreed or expected end of the social interaction.	Late, overtime	Spät, Überstunden	"Work longer than agreed"	"Länger arbeiten als eigentlich vereinbart"	
		**Reason** Specification of by whom or what happened to the current structural course.	**Reason with the Actor** The reason for the current course of social interaction lies with the actor.			"Because of a headache, I left the common ulitmate frisbee game alone, before everyone else"	"Wegen Kopfschmerzen habe ich das gemeinsame Ulitmate Frisbee Spiel allein vor allen anderen verlassen"	

Component	*Level-1 Feature*	*Level-2 Feature*	*Level-3 Feature*	*Examples of Cue Words and Cue Concepts for the Coding[1]*	*Examples of Cue Words and Cue Concepts for the Coding[1] in the Original Study Language German*	*Examples from the Dataset*	*Examples from the Dataset in the Original Study Language German*	*Definition Inspired by[2]*
			**Reason with the Partner** The reason for the current course of social interaction lies with the partner.			"Child came home late, gave some stress"	"Kind kam zu spät nach Hause, gab etwas Stress"	
			**Reason with the Actor and the Partner** The reason for the current course of social interaction lies with both the actor and the partner.					
			**External Circumstances/Factors or Third Party** The reason for the current course of social interaction lies in external circumstances or with third parties.			"Job-related reason for the end of the interaction of my child"	"Arbeitstechnisch bedingte Absage eines Kindes"	
**Evaluation** The final rating of the actor of a social interaction.	**Desirability of the Interaction** Whether the social interaction is welcome or not welcome.	**Desirable** The social interaction is desired and wanted.		Desired, wanted, voluntary, intentional, enjoyment, being friendly, likable	Erwünscht, gewollt, freiwillig, absichtlich, nett, Genuss, freundlich sein, sympathisch	"Sitting comfortably together at dinner, eating well, laughing, having fun"	"Gemütlich beim Abendessen zusammenge-sessen, gut gegessen, gelacht, Spass gemacht"	
		**Undesirable** The social interaction is undesired and unwanted.		Unwanted, must, annoy, prevented, delayed, forced, persuaded, unasked, unfortunately	Unerwünscht, ungewollt, muss, nerven, verhindert, verzögert, Zwang, überredet, ungefragt, leider	"A person who was not so closely connected told me something that was basically of no interest, the conversation was forced on me!"	"Eine nicht so eng verbundene Person mit etwas erzählt hat, das im Grunde für mich nicht von Interesse war, mir wurde das Gespräch aufgezwungen!"	
	**Desirability of the Topic/Content** Whether the topic/content of the	**Desirable** The topic/content of the social		Good news, expression of love, compliments,	Gute Nachrichten, Liebesbekundung, Kompliment, Spass haben,	"laughed at funny videos"	"Über lustige Videos gelacht"	

Component	*Level-1 Feature*	*Level-2 Feature*	*Level-3 Feature*	*Examples of Cue Words and Cue Concepts for the Coding[1]*	*Examples of Cue Words and Cue Concepts for the Coding[1] in the Original Study Language German*	*Examples from the Dataset*	*Examples from the Dataset in the Original Study Language German*	*Definition Inspired by[2]*
	social interaction is welcome or not welcome.	interaction is desired and wanted.		having fun, relief, intention, nodding, affirming, friendly, confirmed, interest, informal, easy	Erleichterung, Absicht, nicken, bejahen, freundlich, bestätigt, Interesse, ungezwungen, locker			
		**Undesirable** The topic/content of the social interaction is undesired unwanted.		Bad news, unwanted topics, lack of interest, refusing a gift, unwanted pity, shaking your head, rolling your eyes, saying no, boring, stupid, annoying, not allowed, prohibited	Schlechte Nachricht, unerwünschte Themen, Desinteresse, Geschenk ablehnen, ungewolltes Mitleid, Kopf schütteln, Augen verdrehen, verneinen, langweilig, blöd, nervig, nicht dürfen, verboten werden	"Information about illness of a friend"	"Information über Erkrankung eines Bekannten"	
	**Valence** The final evaluation of the social interaction regarding positive and negative feelings.	**Positive** The final evaluation of the social interaction is positive.	**Continuously Positive** The entire social interaction is rated as positive.	Positive, nice, pleasant, good	Positiv, schön, angenehm, gut	"I spent a nice morning with my friend"	"Ich verbrachte einen schönen Vormittag mit meinem Freund"	
			**Change from Negative to Positive** During the social interaction there was a shift from negative to positive evaluation of the social interaction.	Problem solving, dispute resolution, successful help, relief	Problemlösung, Streit beheben, erfolgreiche Hilfe, Erleichterung	"A conciliatory conversation with a friend"	"Ein versöhnliches Gespräch mit einer Freundin"	
		**Negative** The final evaluation of the social interaction is negative.	**Continuously Negative** The entire social interaction is rated as negative.	Negative, uncomfortable, bad, not beautiful, not good	Negativ, unangenehm, schlecht, nicht schön, nicht gut	"Unpleasant conversation"	"Unangenehmes Gespräch"	
			**Change from Positive to Negative**	Provoke, irritate	Provozieren, reizen	"One of the guests could not handle	"Eine Person von den Gästen konnte	

Component	*Level-1 Feature*	*Level-2 Feature*	*Level-3 Feature*	*Examples of Cue Words and Cue Concepts for the Coding[1]*	*Examples of Cue Words and Cue Concepts for the Coding[1] in the Original Study Language German*	*Examples from the Dataset*	*Examples from the Dataset in the Original Study Language German*	*Definition Inspired by[2]*
			During the social interaction there was a shift from positive to negative evaluation of the social interaction.			the cozy conversation and tried to argue"	nicht mit den gemütlichen Gesprächen umgehen und versuchte Streit zu machen"	
	**Fulfilment of Expectations** The final evaluation of the social interaction regarding the fulfillment of expectations.	**Realised** Expectations on the social interaction were fulfilled.		Fulfilled	Erfüllt			
		**Not Realised** Expectations on the social interaction were not fulfilled.		Failure to keep promises, frustration	Versprechen nicht einhalten, Frustration	"Just now, first psychologist session. Very nice woman, appointment was much more positive than feared."	"Grad eben, erste Psychologen-Sitzung. Sehr nette Frau, Termin war viel positiver als befürchtet"	

Note. [1]To ensure objectivity, the coding was as close as possible to the specific wording. The cited words and concepts used are based on the probability that the meaning of the description of the social interaction corresponds as closely as possible to the actual coding. Nevertheless, the descriptions have been read separately by the raters and then coded.
[2] The Duden (2019) dictionary served as a support in transliterating terms.

References

Bales, R. F. (1950). A set of categories for the analysis of small group interaction. *American Sociological Review, 15*(2), 257-263. https://doi.org/10.2307/2086790

Bales, R. F., & Strodtbeck, F. L. (1951). Phases in group problem-solving. *The Journal of Abnormal and Social Psychology*, *46*(4), 485-495. https://doi.org/10.1037/h0059886

Barrera, M., Sandler, I. N., & Ramsay, T. B. (1981). Preliminary development of a scale of social support: Studies on college students. *American Journal of Community Psychology*, *9*(4), 435-447.

Bugental, D. B. (2000). Acquisition of the algorithms of social life: A domain-based approach. *Psychological Bulletin*, *126*(2), 187-219. https://doi.org/10.1037/0033-2909.126.2.187

Carstensen, L. L. (1992). Social and emotional patterns in adulthood: support for socioemotional selectivity theory. *Psychology and Aging*, *7*(3), 331-338. https://doi.org/10.1037/0882-7974.7.3.331

Carstensen, L. L., Isaacowitz, D. M., & Charles, S. T. (1999). Taking time seriously: A theory of socioemotional selectivity. *American Psychologist*, *54*(3), 165-181. https://doi.org/10.1037/0003-066X.54.3.165

Cialdini, R. B., Brown, S. L., Lewis, B. P., Luce, C., & Neuberg, S. L. (1997). Reinterpreting the empathy–altruism relationship: When one into one equals oneness. *Journal of Personality and Social Psychology*, *73*(3), 481-494. https://doi.org/10.1037/0022-3514.73.3.481

Deci, E. L., & Ryan, R. M. (2000). The" what" and" why" of goal pursuits: Human needs and the self-determination of behavior. *Psychological Inquiry*, *11*(4), 227-268. https://doi.org/10.1207/S15327965PLI1104_01

Deci, E. L., & Ryan, R. M. (2004). *Handbook of self-determination research*. University Rochester Press.

Duckworth, K. L., Bargh, J. A., Garcia, M., & Chaiken, S. (2002). The automatic evaluation of novel stimuli. *Psychological Science*, *13*(6), 513-519. https://doi.org/10.1111/1467-9280.00490

Dudenredaktion (2019). *Duden – The german orthography*. Bibliographic Institute.

Eckes, T. (1995). Features of situations: A two-mode clustering study of situation prototypes. *Personality and Social Psychology Bulletin*, *21*(4), 366-374. https://doi.org/10.1177/0146167295214007

Ekman, P (1992). Are there basic emotions? *Psychological Review, 99*(3), 550-553. https://doi.org/10.1037/0033-295X.99.3.550

Fisherl, C. D. (1993). Boredom at work: A neglected concept. *Human Relations*, *46*(3), 395-417. https://doi.org/10.1177/001872679304600305

Folkman, S., & Lazarus, R. S. (1984). *Stress, appraisal, and coping* (p. 460). New York: Springer Publishing Company.

Forgas, J. P. (1976.) The perception of social episodes: categorical and dimensional representations in two different social milieus. *Journal of Personality and Social Psychology, 32*, 199-09. https://doi.org/10.1016/B978-0-08-023719-0.50015-2

Fredrickson, B. L. (2004). The broaden–and–build theory of positive emotions. *Philosophical Transactions of the Royal Society of London. Series B: Biological Sciences*, *359*(1449), 1367-1377. https://doi.org/10.1098/rstb.2004.1512

Gable, S. L., & Berkman, E. T. (2008). Making connections and avoiding loneliness: Approach and avoidance social motives and goals. In A. J. Elliot (Ed.), *Handbook of Approach and Avoidance Motivation* (pp. 204–216). New York, NY: Psychology Press.

Goffman, E. (1974). *Frame analysis: An essay on the organization of experience*. Harvard University Press.

Hamp, B., & Feldweg, H. (1997). Germanet-a lexical-semantic net for german. *Automatic Information Extraction and Building of Lexical Semantic Resources for NLP Applications,* 9-15.

Henrich, V., & Hinrichs, E. (2010). *GernEdiT - The GermaNet Editing Tool.* The Seventh Conference on International Language Resources and Evaluation. http://lrec-conf.org/lrec2010/

Hess, U., Adams Jr, R., & Kleck, R. (2005). Who may frown and who should smile? Dominance, affiliation, and the display of happiness and anger. *Cognition and Emotion*, *19*(4), 515-536. https://doi.org/10.1080/02699930441000364

Horwitz, A. V, McLaughlin, J., & White, H. R. (1997). How the negative and positive aspects of partner relationships affect the mental

health of young married people. *Journal of Health and Social Behavior*, *39*(2), 124-136. https://doi.org/10.2307/2676395

House, J. S., & Kahn, R. L. (1985). Measures and concepts of social support. In S. Cohen & S.L. Syme (Eds.), *Social Support and Health* (pp. 83-108). New York: Academic Press.

Ingersoll-Dayton, B., Morgan, D., & Antonucci, T. (1997). The effects of positive and negative social exchanges on aging adults. *Journals of Gerontology - Series B Psychological Sciences and Social Sciences*, *52*(4), 190-199. https://doi.org/10.1093/geronb/52B.4.S190

Kasser, T. & Ryan, R. M. (1993). A dark side of the American dream: Correlates of financial success as a central life aspiration. *Journal of Personality and Social Psychology, 65*(2), 410-422.
https://doi.org/0.1037/0022-3514.65.2.410

King, G. A., & Sorrentino, R. M. (1983). Psychological dimensions of goal-oriented interpersonal situations. *Journal of Personality and Social Psychology*, *44*(1), 140-162. https://doi.org/10.1037/0022-3514.44.1.140

Krause, M. S. (1970). Use of social situations for research purposes. *American Psychologist*, *25*(8), 748-753.
https://doi.org/10.1037/h0029822

Krause, N. (1995). Negative interaction and satisfaction with social support among older adults. *Journals of Gerontology - Series B Psychological Sciences and Social Sciences*, *50 B*(2), 59-73. https://doi.org/10.1093/geronb/50B.2.P59

Krause, N., & Markides, K.S. (1990). Measuring social support among older adults. *International Journal of Aging and Human Development, 30,* 37-53.

Lakey, B., Tardiff, T. A., & Drew, J. B. (1994). Negative social interactions: Assessment and relations to social support, cognition, and psychological distress. *Journal of Social and Clinical Psychology*, *13*(1), 42-62. https://doi.org/10.1521/jscp.1994.13.1.42

Lewin, K. (1936). *Principles of topological psychology*. York.

Lodi-Smith, J., & Roberts, B. W. (2007). Social investment and personality: A meta-analysis of the relationship of personality traits to investment in work, family, religion, and volunteerism. *Personality and Social Psychology Review*, *11*(1), 68-86.
https://doi.org/10.1177/1088868306294590

Magnusson, D. (1971). An analysis of situational dimensions. *Perceptual and Motor Skills*, *32*(3), 851-867.

https://doi.org/10.2466/pms.1971.32.3.851

Marcatto, F., & Ferrante, D. (2008). The regret and disappointment scale: An instrument for assessing regret and disappointment in decision making. *Judgment and Decision Making*, *3*(1), 87-99.

Maslow, A. H. (1970). *Motivation and personality*. New York: Harper and Row.

Maslow, A. H. (1943). A theory of human motivation. *Psychological Review, 50*(4), 370-96. https://doi.org/10.1037/h0054346

Maslow, A. H. (1954). *Motivation and personality*. New York: Harper and Row.

McClelland, D. C. (1988). *Human motivation*. Cambridge University Press.

McClelland, D. C., Atkinson, J. W., Clark, R. A., & Lowell, E. L. (1953). *The achievement motive*. New York: Appleton-Century-Crofts.

Mehrabian, A., & Ksionzky, S. (1974). *A theory of affiliation*. Lexington, KY: Heath.

Moos, R. H. (1973). Conceptualizations of human environments. *American Psychologist*, *28*(8), 652-665. https://doi.org/10.1037/h0035722

Nascimento-Schulze, C. M. (1981). Towards situational classification. *European Journal of Social Psychology*, *11*(2), 149-159. https://doi.org/10.1002/ejsp.2420110203

Newsom, J. T., Nishishiba, M., Morgan, D. L., & Rook, K. S. (2003). The relative importance of three domains of positive and negative social exchanges: a longitudinal model with comparable measures. *Psychology and Aging*, *18*(4), 746-754. https://doi.org/10.1037/0882-7974.18.4.746

Newsom, J. T., Rook, K. S., Nishishiba, M., Sorkin, D. H., & Mahan, T. L. (2005). Understanding the relative importance of positive and negative social exchanges: Examining specific domains and appraisals. *Journals of Gerontology - Series B Psychological Sciences and Social Sciences*, *60*(6), 304-312. https://doi.org/10.1093/geronb/60.6.P304

O'Connor, M. F., & Sussman, T. J. (2014). Developing the yearning in situations of loss scale: Convergent and discriminant validity for bereavement, romantic breakup, and homesickness. *Death Studies*, *38*(7), 450-458. https://doi.org/10.1080/07481187.2013.782928

Okun, M. A., & Keith, V. M. (1998). Effects of positive and negative social exchanges with various sources on depressive symptoms in younger and older adults. *Journals of Gerontology - Series B Psychological Sciences and Social Sciences*, *53*(1). https://doi.org/10.1093/geronb/53B.1.P4

Oreg, S., Edwards, J. A., & Rauthmann, J. F. (2020). The situation six: Uncovering six basic dimensions of psychological situations from the Hebrew language. *Journal of Personality and Social Psychology*, *118*(4), 835-863. https://doi.org/10.1037/pspp0000280

Ortony, A., & Turner, T. J. (1990). What's basic about basic emotions? *Psychological Review*, *97*(3), 315-331. https://doi.org/10.1037/0033-295X.97.3.315

Parrigon, S., Woo, S. E., Tay, L., & Wang, T. (2017). CAPTION-ing the situation: A lexically-derived taxonomy of psychological situation characteristics. *Journal of Personality and Social Psychology*, *112*(4), 642-681. https://doi.org/10.1037/pspp0000111

Perkins, R. E., & Hill, A. B. (1985). Cognitive and affective aspects of boredom. *British Journal of Psychology*, *76*(2), 221-234. https://doi.org/10.1111/j.2044-8295.1985.tb01946.x

Price, R. H., & Blashfield, R. K. (1975). Explorations in the taxonomy of behavior settings - Analysis of dimensions and classification of settings. *American Journal of Community Psychology*, *3*(4), 335-351. https://doi.org/10.1007/BF00880776

Rauktis, M. E., Koeske, G. F., & Tereshko, O. (1995). Negative social interactions, distress, and depression among those caring for a seriously and persistently mentally III relative. *American Journal of Community Psychology*, *23*(2), 279-299. https://doi.org/10.1007/BF02506939

Rauthmann, J. F., Gallardo-Pujol, D., Guillaume, E. M., Todd, E., Nave, C. S., Sherman, R. A., Ziegler, M., Jones, A. B., & Funder, D. C. (2014). The situational Eight DIAMONDS: A taxonomy of major dimensions of situation characteristics. *Journal of Personality and Social Psychology*, *107*(4), 677-718. https://doi.org/10.1037/a0037250

Reis, H. T., Collins, W. A., & Berscheid, E. (2000). The relationship context of human behavior and development. *Psychological Bulletin*, *126*(6), 844-872. https://doi.org/10.1037/0033-2909.126.6.844

Reis, H. T., & Wheeler, L. (1991). Studying social interaction with the Rochester Interaction Record. In *Advances in Experimental Social Psychology* (Vol. 24, pp. 269-318). Academic Press.

Roberts, B. W., & Wood, D. (2006). Personality development in the context of the neo-socioanalytic model of personality. In D. Mroczek &

T. Little (Eds.), *Handbook of Personality Development*. Mahwah, NJ: Lawrence Erlbaum.

Roberts, B. W., Wood, D., & Smith, J. L. (2005). Evaluating five factor theory and social investment perspectives on personality trait development. *Journal of Research in Personality*, *39*(1), 166-184. https://doi.org/10.1016/j.jrp.2004.08.002

Rook, K. S. (2001). Emotional health and positive versus negative social exchanges: A daily diary analysis. *Applied Developmental Science*, *5*(2), 86-97. https://doi.org/10.1207/S1532480XADS0502_4

Rook, K., & Pietromonaco, P. (1987). Close relationships: Ties that heal or bind? In W. H. Jones & D. Perlman (Eds.), *Advances in Personal Relationships: A Research Annual* (pp. 1-35). Greenwick: JAI.

Rose, A., Peters, N., Shea, J. A., & Armstrong, K. (2004). Development and testing of the health care system distrust scale. *Journal of General Internal Medicine*, *19*(1), 57-63.

Ruehlman, L. S., & Karoly, P. (1991). With a Little Flak From My Friends: Development and preliminary validation of the test of negative social exchange (TENSE). *Psychological Assessment*, *3*(1), 97-104.
https://doi.org/10.1037/1040-3590.3.1.97

Russell, D., Peplau, L. A., & Cutrona, C. E. (1980). The revised UCLA loneliness scale: concurrent and discriminant validity evidence. *Journal of Personality and Social Psychology*, *39*(3), 472. http://dx.doi.org/ 10.1037/0022-3514.39.3.472

Sääksjärvi, M., & Morel, K. P. (2010). The development of a scale to measure consumer doubt toward new products. *European Journal of Innovation Management, 13*(3), 272-293. http://dx.doi.org/10.1108/14601061011060120

Saucier, G., Bel-Bahar, T., & Fernandez, C. (2007). What modifies the expression of personality tendencies? defining basic domains of situation variables. *Journal of Personality*, *75*(3), 479-504. https://doi.org/10.1111/j.1467-6494.2007.00446.x

Schulz, R., Williamson, G. M., Morycz, R. K., & Biegel, D. E. (1992). Costs and benefits of providing care to Alzheimer's patients. In S. Spacapan, & S. Oskamp (Eds.), *Helping and Being Helped: Naturalistic Studies, Claremont Symposium on Applied Social Psychology* (pp. 153-181). Newbury Park, CA: Sage.

Schuster, T. L., Kessler, R. C., & Aseltine, R. H. (1990). Supportive interactions, negative interactions, and depressed mood. *American Journal of Community Psychology*, *18*(3), 423-438. https://doi.org/10.1007/BF00938116

Schwartz, S. H., & Bardi, A. (2001). Value hierarchies across cultures: Taking a similarities perspective. *Journal of Cross-Cultural Psychology*, *32*(3), 268-290. https://doi.org/10.1177/0022022101032003002

Schwarzer, R., & Schulz, U. (2000). *Berlin Social Support Scales (BSSS).*

Sherman, R. A., Nave, C. S., & Funder, D. C. (2012). Properties of persons and situations related to overall and distinctive personality-behavior congruence. *Journal of Research in Personality*, *46*(1), 87-101. https://doi.org/10.1016/j.jrp.2011.12.006

Sherman, R. A., Nave, C. S., & Funder, D. C. (2013). Situational construal is related to personality and gender. *Journal of Research in Personality*, *47*(1), 1-14. https://doi.org/10.1016/j.jrp.2012.10.008

Swiss Civil Code (1907, December 10).

Stafford, M., McMunn, A., Zaninotto, P., & Nazroo, J. (2011). Positive and negative exchanges in social relationships as predictors of depression: Evidence from the English Longitudinal Study of Aging. *Journal of Aging and Health*, *23*(4), 607-628. https://doi.org/10.1177/0898264310392992

Thomson, D. M., & Crocker, C. (2013). A data-driven classification of feelings. *Food Quality and Preference*, *27*(2), 137-152. https://doi.org/10.1016/j.foodqual.2012.09.002

Van Heck, G. L. (1984). The construction of a general taxonomy of situations. In H. Bonarius, G. L.

Van Heck, G. L. (1989). Situation concepts: Definitions and classification. In P. J. Hettema (Ed.), *Personality and Environment: Assessment of Human Adaptation* (pp. 53-69). Oxford, England: Wiley.

Wyatt, F. (1947). The scoring and analysis of the Thematic Apperception Test. *The Journal of Psychology*, *24*(2), 319-330. https://doi.org/10.1080/00223980.1947.9917359

Zinck, A., & Newen, A. (2008). Classifying emotion: A developmental account. *Synthese*, *161*(1), 1-25. https://doi.org/10.1007/s11229-006-9149-2
